# Inactivation of siderophore iron‐chelating moieties by the fungal wheat root symbiont *Pyrenophora biseptata*

**DOI:** 10.1111/1758-2229.13234

**Published:** 2024-01-19

**Authors:** Katie S. French, Emmanuel Chukwuma, Ilan Linshitz, Kosuke Namba, Owen W. Duckworth, Marc A. Cubeta, Oliver Baars

**Affiliations:** ^1^ Department of Entomology and Plant Pathology North Carolina State University, Center for Integrated Fungal Research Raleigh North Carolina USA; ^2^ Department of Chemistry North Carolina State University Raleigh North Carolina USA; ^3^ Department of Biology University of Maryland College Park Maryland USA; ^4^ Department of Pharmaceutical Sciences Tokushima University Tokushima Japan; ^5^ Department of Crop and Soil Sciences North Carolina State University Raleigh North Carolina USA; ^6^ Present address: Department of Soil Science University of Arkansas Fayetteville Arkansas USA

## Abstract

We investigated the ability of four plant and soil‐associated fungi to modify or degrade siderophore structures leading to reduced siderophore iron‐affinity in iron‐limited and iron‐replete cultures. *Pyrenophora biseptata*, a melanized fungus from wheat roots, was effective in inactivating siderophore iron‐chelating moieties. In the supernatant solution, the tris‐hydroxamate siderophore desferrioxamine B (DFOB) underwent a stepwise reduction of the three hydroxamate groups in DFOB to amides leading to a progressive loss in iron affinity. A mechanism is suggested based on the formation of transient ferrous iron followed by reduction of the siderophore hydroxamate groups during fungal high‐affinity reductive iron uptake. *P. biseptata* also produced its own tris‐hydroxamate siderophores (neocoprogen I and II, coprogen and dimerum acid) in iron‐limited media and we observed loss of hydroxamate chelating groups during incubation in a manner analogous to DFOB. A redox‐based reaction was also involved with the tris‐catecholate siderophore protochelin in which oxidation of the catechol groups to quinones was observed. The new siderophore inactivating activity of the wheat symbiont *P. biseptata* is potentially widespread among fungi with implications for the availability of iron to plants and the surrounding microbiome in siderophore‐rich environments.

## Graphical abstract

The wheat root symbiont fungus *Pyrenophora biseptata* degrades iron‐chelating groups in extracellular siderophores, forming weaker and more labile iron complexes. As a result, iron is expected to become available to plants and microbes that do not possess specialized siderophore uptake mechanisms.

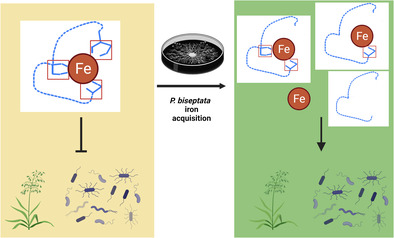

## INTRODUCTION

Iron is an essential but poorly available micronutrient needed as a cofactor and catalyst in many cellular and enzymatic processes. Siderophores are specialized metabolites produced and secreted by bacteria, fungi, and graminaceous plants when iron bioavailability is low. These secretions enhance iron bioavailability by binding, solubilizing and transporting biologically unavailable ferric iron (Fe^3+^), such as iron‐bound in iron oxides or humic matter. Siderophores are recognized as key agents of competition and cooperation in microbial communities and host‐microbe‐mediated interactions (Kramer et al., 2020; Winkelmann, 2007). The ability to interfere with siderophore iron acquisition could provide a biological mechanism to gain a competitive advantage in microbial and plant community interactions, for example, by reducing iron affinity via structural modifications. Organisms may also degrade siderophores to metabolize them as carbon substrates.

In general, siderophores are thought to exhibit high stability towards biological degradation because of chemical modifications that protect from enzymatic degradation and because iron chelation can hinder the attack of hydrolytic enzymes (Winkelmann et al., 1999). However, a few studies have previously observed microbial degradation of siderophores when growing bacteria with siderophores as a carbon source (Warren & Neilands, 1964; Winkelmann et al., 1999; Zaya et al., 1998). In these studies, siderophores were only degraded when present in their free (apo) form, not bound to iron. In addition, several studies have shown the degradation of siderophore structures as part of a cellular mechanism to recover iron from siderophores: fungal esterases are known to be responsible for the hydrolysis of iron‐bound fusarinines (Ecker et al., 2018; Gründlinger et al., 2013; Kragl et al., 2007) and bacterial esterases can hydrolyze enterobactin and salmochelin iron complexes (Caza et al., 2015; Perraud et al., 2018; Zeng et al., 2013). The siderophore hydrolysis products have lower iron affinity and promote cellular iron utilization via ligand exchange or iron reduction. Siderophore degradation products, such as mono‐ and bis‐hydroxamates from the hydrolysis of fusarinines also make iron more available to plants (Hördt et al., 2000). In contrast, the unmodified tris‐hydroxamate siderophore desferrioxamine B (DFOB) was previously reported to be not available to maize and oat plants and provided a competitive advantage to bacteria able to utilize this siderophore for Fe acquisition (Bar‐Ness et al., 1992). The degradation or structural modification of siderophores by bacteria or fungi could thus play a critical role in iron acquisition by plants and non‐siderophore‐producing microbes. In the following, we will use the term ‘degradation’ to describe any siderophore reactions leading to products with structural modifications that have reduced iron affinity.

**FIGURE 1:**
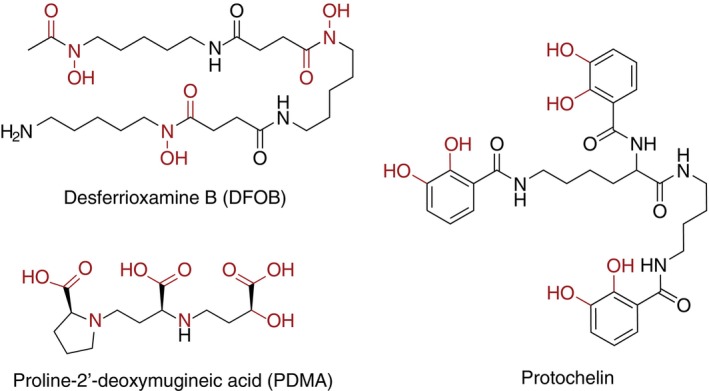
Structures of model siderophores used in the degradation experiments. The different moieties involved in Fe complexation are indicated in red.

In this study, we followed siderophore production and degradation by four plant and soil‐associated fungi with varying ecology and substrate preference: *Phanerodontia* (=*Phanerochaete) chrysosporium* RP‐78, *Phanerodontia chrysosporium* BKM‐1767, *Pyrenophora* (=*Drechslera*) *biseptata* and *Linnemannia* (=*Mortierella*) *elongata* 624. Three representative siderophore standards were chosen, representing natural diversity in siderophore structures and iron‐chelating groups: the tris‐hydroxamate siderophore DFOB, the tris‐catecholamide siderophore protochelin, and the carboxylate phytosiderophore proline‐2′‐deoxymugineic acid (PDMA) (Figure 1). DFOB and protochelin are bacterial siderophores and PDMA is a synthetic analog of a plant siderophore that has been introduced as plant iron‐fertilizer (Suzuki et al., 2021). In addition to analyses of the degradation of these three siderophore models, we investigated the production of fungal siderophores. The results are discussed in terms of the potential of fungi to shape siderophore dynamics in plant interactions with important consequences for iron acquisition by plants and plant microbiome members.

## EXPERIMENTAL PROCEDURES

### Fungal isolates

Four species of fungi were selected for this study: two different strains of a white rot wood decay fungus—(1) *Phanerodontia* (=*Phanerochaete*) *chrysosporium* RP‐78, and (2) *Phanerodontia chrysosporium* BKM‐1767 (Hjortstam & Ryvarden); a wheat root associated symbiont—(3) *Pyrenophora* (=*Drechslera*) *biseptata* (Sacc. & Roum.) and a root endophyte isolated from eastern cottonwood—(4) *Linnemannia* (=*Mortierella*) *elongata* (Vandepol & Bonito) (Bonito et al., 2014). The strain of *Linnemania elongata* was supplied by Jessie Uehling (Oregon State University) and the two strains of *P. chrysosporium* were supplied by Dan Cullen (US Department of Agriculture Forest Products Laboratory, Madison, WI). *P. biseptata* was isolated from wheat roots in our laboratory. *L. elongata* was originally isolated from *P. deltoides* roots in North Carolina and a culture was available in our laboratory (Becker & Cubeta, 2020). Isolates were maintained by adding 5‐mm diameter mycelial plugs taken from an actively growing colony to 0.5 mL of a mixture of sterile 50% glycerol and 0.5 mL potato dextrose broth (Difco) in cryovials. The cryovials were incubated for 24 h at 25°C and stored at −80°C. Fungi were revived by transferring mycelial plugs to potato dextrose agar (PDA; Difco) plates.

### Siderophore standards

The siderophores were selected to represent the three most common iron binding groups found in siderophores: the trishydroxamate siderophore DFOB (mesylate salt, MilliporeSigma), the triscatecholate siderophore protochelin and the carboxylate siderophore proline‐2′‐deoxymugineic acid (PDMA) (Figure 1). Protochelin was synthetically prepared by the Small Molecule Synthesis Facility at Duke University and used as received. PDMA was kindly provided as PDMA∙2HCl by Aichi Steel Corporation (Japan) (Suzuki et al., 2021). Stock solutions of 10 mM were prepared in methanol for protochelin or 18‐MΩ‐cm water (MQ water) for DFOB and PDMA, respectively. A 10‐mM standard of iron‐bound DFOB (FeDFOB) was prepared by the addition of 10 mM FeCl_3_ to a 10 mM solution of DFOB in MQ water. Siderophore stocks were immediately used or stored for up to 2 weeks at −20°C.

### Preparation of fungal mycelium and growth media

Mycelial plugs (5 mm diameter) were taken from the edge of an actively growing culture on PDA plates. The mycelial plugs were then placed on 2% malt extract agar (Difco) and grown for 7 days at 25°C. A new mycelial plug was taken from this culture and placed on a PDA agar plate (9 cm diameter) at 25°C until the mycelial growth of the fungus reached the edge of the plate, for approximately 7 days. A mycelial plug from each isolate was then transferred to 1.5% malt extract broth (Difco) and incubated for 3–5 days. The cultures were then harvested by vacuum filtration with cellulose filters (Whatman #1, 11 μm), rinsed with sterile MQ water, and homogenized with a handheld homogenizer (Fisher Scientific 150) using a 7‐mm probe for 1 min on low speed to achieve a homogenous inoculum for the siderophore reactions. Experiments were started by adding 2 mL of the homogenized mycelia to 13 mL of minimal salt broth (MSB) growth medium in sterile cell culture flasks (Nunc, 75 cm^2^). The MSB medium was slightly modified from a previously reported medium used to stimulate the production of ligninolytic enzymes by *Phlebia floridensis* (Arora & Gill, 2005). A volume of 1 L of MSB medium was prepared by dissolving 10 g glucose, 2 g KH_2_PO_4_, 0.5 g MgSO_4_∙7H_2_O, 0.1 g CaCl_2_∙2H_2_O, 1 mg thiamine HCl, 0.2 g ammonium tartrate, 1 mL trace metal solution containing [Zn^2+^] = 53 μM, [Cu^2+^] = 10 μM, [Co^2+^] = 24 μM, [Mn^2+^] = 225 μM, [MoO_4_^2−^] = 100 μM, [BO_3_^3−^] = 10 mM) and 1 mL EDTA (0.1 M) in MQ water and adjusting the pH = 4.5 ± 0.1 with NaOH and H_2_SO_4_. The medium was then filter sterilized (0.2 μm, PES, ThermoFisher) before use. Media were used as is (iron‐deficient medium) or 300 μM FeCl_3_ was added to the medium prior to inoculation (iron‐replete media). Immediately after inoculation, a final concentration of 6.67 μM of each siderophore (10 μL of the 10 mM stock solutions) was added to the 15 mL culture flasks.

### Siderophore degradation experiments

Three experiments were conducted. *Experiment 1* had the purpose of comparing the ability of each of the four fungi to degrade each of the three siderophore structures. One replicate of each culture was incubated in MSB medium containing siderophores without and with added iron. Sterile MSB medium containing siderophores was used as a positive control. Samples were collected after an incubation time of 48 h. Supernatant and fungal cells were separated by centrifugation. The supernatant was further filtered using a syringe filter (0.2 μm polyethersulfone, PES, membrane) and stored at −80°C before analysis by LC–MS (see details below). The mycelium of each fungus was harvested by vacuum filtration (Whatman #1, 11 μm), rinsed with sterile 18 MΩ water (MQ water) and lyophilized to determine fungal biomass. This experiment indicated *Pyrenophore biseptata* was highly effective in degrading DFOB and protochelin in contrast to the *Linnemannia elongata* and *Phanerodontia chrysosporium*. Therefore, a second experiment (Experiment 2) focused on replicating this result and taking additional time points with triplicates of *P. bispetata* in iron‐limited MSB media with DFOB and protochelin. Supernatant samples were collected immediately after inoculation, after 1 day, and after 3 days by filtration (0.2 μm PES syringe filters). A sterile control containing siderophores was also set up at the same time as the incubations. The outcome of these first two experiments suggested that iron chelation protects DFOB from degradation by *P. biseptata*. To confirm this, *Experiment 3* was set up in which *P. biseptata* was cultured with unbound DFOB and compared to incubations with DFOB bound to Fe (FeDFOB). These experiments were done in a modified MSB medium from which EDTA and the trace metal solution were omitted from the medium (see above). The incubation with unbound DFOB was done without any added iron in the medium, and the incubation with added FeDFOB was done with the addition of 100 μM FeCl_3_. Culture supernatants were collected after 5 days by filtration (0.2 μm PES syringe filters).

### Analysis of siderophore degradation, formation of degradation products, and siderophore production by LC–MS

All fungal culture supernatants were analysed by liquid chromatography–mass spectrometry (LC–MS) using an ISQ‐EC (ThermoFisher) single quadrupole mass spectrometer in combination with an Ultraviolet–visible (UV–vis) spectrophotometer and a Charged Aerosol Detector (ThermoFisher). A sample volume of 25 μL was injected and separated under a gradient of solvents A and B (unless otherwise noted A: water, 0.1% formic acid, 1% acetonitrile; B: acetonitrile, 0.1% formic acid, 2% water; gradient: 0–1.5 min 0% B, 1.5–8 min 0%–100% B; 8–10 min 100% B; re‐equilibration at 100% A for 4 min). Separation was accomplished with an Agilent Poroshell 120 EC‐C18 column (4.6 × 100 mm^2^, 2.7 μm) and a flow rate of 1.2 mL/min, and the column temperature was 30°C. The ISQ‐EC mass spectrometer was operated in single ion monitoring mode targeting the three added siderophores and their degradation products (Table S1). Samples from Experiment 2 were additionally analysed with a high‐resolution liquid chromatography–tandem mass spectrometry (LC–MS/MS) platform (Orbitrap Exploris 480, ThermoFisher). Sample volumes of 25 μL were injected and separated using a Restek Raptor C18 (2.1 × 100) column with a flow rate of 0.4 mL/min and the column compartment was kept at 45°C. The mobile phase gradient was identical to that used with the ISQ‐EC described above. Full‐scan mass spectra (*m/z* = 85–1200) were acquired in positive ionization mode with the resolution set to *R* = 60,000 (full width at half maximum at *m/z* = 400) and data‐dependent MS/MS acquisition of the top five most abundant ions in each cycle. Product ions were generated in HCD mode with 35 eV collision energy and an isolation window of 1.5 Da. The resolving power for MS/MS analysis was *R* = 15,000 (full width at half maximum at *m/z* = 400). Siderophore degradation was followed by tracking peak areas of the three siderophores added. Putative degradation products of the added siderophore standards were identified in high‐resolution LC–MS data by searching for new LC–MS peaks in incubated samples with related *m/z* values (e.g., simple mass differences, such as –O; similar mass defects) and MS/MS spectra (e.g., presence of 2,3‐dihydroxybenzoic acid moieties in protochelin degradation products showing as neutral loss of *m/z* = 136.016 in MS/MS spectra; Baars & David, 2016). Analysis of protochelin degradation products was also done utilising LC–UV–vis by looking for new peaks with absorption maxima in the range of the absorption maxima of the 2,3‐dihydroxybenzoic acid groups in protochelin (*λ* = 315 nm; Baars et al., 2015).   Untargeted siderophore analysis was done using high‐resolution LC–MS/MS by searching for the specific ^54^Fe‐^56^Fe isotope pattern associated with Fe‐siderophore complexes, along with searching for the specific mass difference between the apo‐siderophore and the Fe‐siderophore complex and siderophore‐characteristic MS/MS fragmentation patterns as (Baars et al., 2014; Baars & David, 2016). In addition, the production of fungal siderophores was assayed using the Chrome Azurol S (CAS) assay (Smith, 1998).

### *Enzyme extraction from* P. biseptata *cell‐pellets and siderophore degradation assays*

The mycelium of the fungus was grown in MSB medium with and without added iron, as described for Experiment 1 (see section ‘Siderophore degradation experiments’ above). The supernatant and cell pellet were separated by centrifugation. The supernatant was sterile‐filtered (0.2 μM PES membrane syringe filter) for assays of siderophore degradation. Crude cell‐pellet enzyme extracts for assays of siderophore degradation by intracellular enzymes were prepared using a protocol previously reported for mycelium of the fungus *Magnaporthe oryzae* (Oh et al., 2012). Cell pellets were freeze‐dried and 500 mg of freeze‐dried sample was ground using a mortar and pestle with liquid nitrogen. Samples were resuspended with 2 mL of a lysis buffer containing 50 mM HEPES (pH = 7.5), 0.5% Nonidet P‐40, 250 mM NaCl, 10% (v/v) glycerol, 2 mM EDTA (pH 8.0) and a protease inhibitor cocktail (cOmplete™, Roche) (Oh et al., 2012). To assay for siderophore degradation, a concentration of 20 μM of DFOB, FeDFOB or protochelin was added to 5 mL of liquid supernatant or 5 mL MSB before incubating for 3 days at 100 rpm. Samples were then filtered (0.2 μm) and analyzed by LCMS.

## RESULTS

### Decrease of dissolved siderophore concentrations during fungal incubations

**FIGURE 2:**
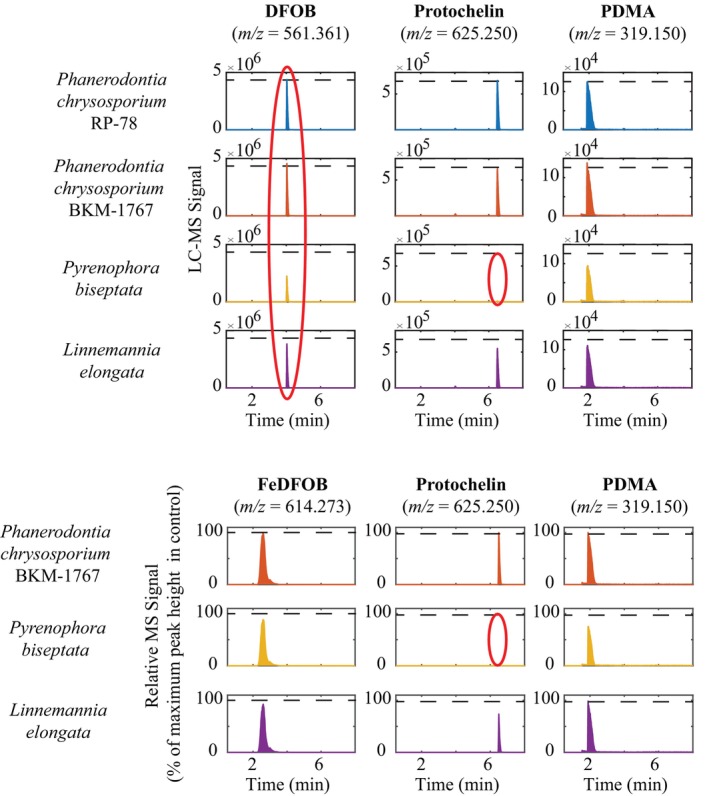
LC–MS peaks showing dissolved siderophores after incubation in iron‐limited MSB media for each of the four mycelial slurries after 2 days in comparison to initial peak heights (dashed line). LC–MS, liquid chromatography–mass spectrometry; MSB, minimal salt broth.

The mycelium of each of the four fungi was incubated with the three siderophores added at a concentration of 6.67 μM in MSB medium. The incubation time was 2 days in iron‐limited (Figure 2) and iron‐replete MSB media (Figure S1). We found that the activity of *P. biseptata* leads to an approximately 50% decrease in DFOB concentrations in iron‐limited medium (Figure 2) in contrast to the two strains of *P. chrysosporium* and *L. elongata*, which did not change DFOB concentrations significantly. For protochelin, the siderophore was almost completely removed from the solution with *P. biseptata*, whereas the two *Phanerodontia* strains and *L. elongata* showed no or minor changes in dissolved protochelin concentrations. PDMA concentrations were less affected by *P. biseptata* compared to DFOB and protochelin, with a ~30% decrease in solution. The other fungi showed no (*P. chrysosprium* strains) or minor (*L. elongata*) ability to decrease dissolved concentrations of this siderophore. Comparable results were obtained with incubations in iron‐replete MSB medium (Figure S1) except in the case of DFOB, for which the concentration decrease was much smaller with *P. biseptata* (~15%). Taken together, these results suggested a specialized ability of *P. biseptata* to remove diverse siderophore structures from the solution. The presence of iron reduced the ability of *P. biseptata* to remove DFOB from the solution.

### *Degradation of DFOB by* P. biseptata

**FIGURE 3:**
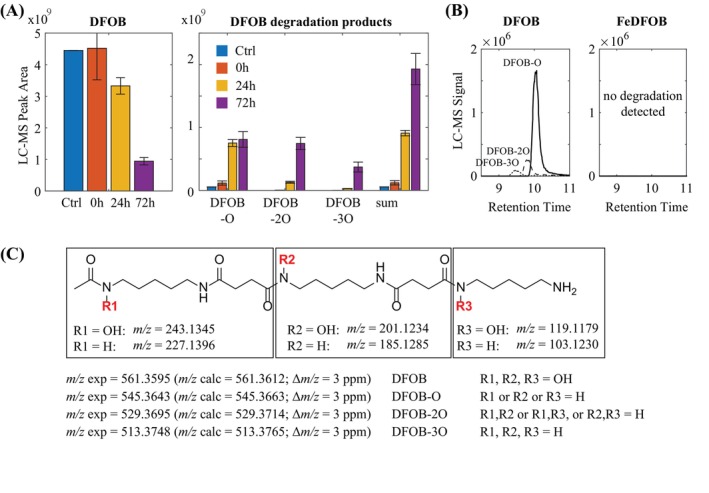
Degradation of DFOB by *P. biseptata*. (A) LC–MS peak areas for DFOB and DFOB degradation products for each of the three time points. Measurements of DFOB and DFOB degradation products were done simultaneously in the same samples. Error bars indicate standard deviations from triplicate biological incubations. (B) LC–MS chromatograms for the three degradation products after 5 days incubation in iron‐limited media with DFOB and iron‐replete media with FeDFOB. (C) Structures of the observed DFOB degradation products derived from tandem mass‐spectrometry fragmentation spectra. Fragments observed in MS/MS spectra (see also Table S2) are shown and correspond to the intact iron‐chelating hydroxamates or to the corresponding structures in which hydroxamates were reduced to amides. DFOB, desferrioxamine B; LC–MS, liquid chromatography–mass spectrometry.

To confirm the above results and further investigate potential degradation products and mechanisms, a second incubation experiment was conducted with *P. biseptata* where supernatants from triplicate cultures in iron‐limited MSB medium were sampled at three timepoints (immediately after inoculation, 24 and 72 h) and subsequently analyzed by high‐resolution LC–MS. The results confirmed a significant decrease in dissolved DFOB concentrations (~25% after 24 h and ~70% after 72 h) and, at the same time, the formation of putative DFOB degradation products was observed (Figure 3A). The three main observed degradation products had *m/z* values in agreement with the sum formulas of DFOB less than one to three oxygen atoms (DFOB‐O, DFOB‐2O and DFOB‐3O). The time points were consistent with DFOB‐O formed first, followed by DFOB‐2O, and finally DFOB‐3O. The relatively minor modifications of the DFOB structure were not expected to strongly impact ionization behavior in LC‐ESI‐MS. Therefore, the peak area sum of the degradation products was used to estimate whether most of the DFOB concentration decrease in solution could be explained by the formation of these three degradation products. After 24 and 72 h, the peak area sum of the three degradation products accounted for ~80% and ~55% of the dissolved DFOB loss, respectively. This good agreement between DFOB peak area decrease and increase in peak areas of DFOB degradation products suggested that the major fraction of DFOB reacted in the supernatant solution and was not lost via sorption to cell surfaces or sequestered as substrates.

MS/MS spectra showed fragmentation of the three degradation products consistent with DFOB in which one, two or three hydroxamate groups were reduced to amides (Figure 3C, Table S2). Fragmentation spectra show that the reduction of the hydroxamate groups in DFOB‐O occurred in any of the three hydroxamate positions leading to three degradation products that were not chromatographically separated (Figure 3C, Table S2). The same was true for DFOB‐2O in which any two of the three hydroxamate groups were reduced to amides. In the final degradation product, DFOB‐3O, all three hydroxamate moieties were reduced to amides. Because the three hydroxamate moieties are the iron chelating groups in DFOB, their loss is expected to lead to a loss of iron chelating ability in the degradation products. Indeed, a fraction of DFOB was bound to iron under our LC–MS conditions, as shown by an LC–MS peak corresponding to FeDFOB. In contrast, DFOB‐3O did not show any corresponding Fe chelates in LC–MS. DFOB‐O and DFOB‐2O showed increasingly lower peak area fractions of Fe chelates compared to the respective apo siderophores. It should be noted that the analysis of Fe chelation via LC–MS does not equate to the speciation of DFOB and DFOB degradation products in the culture media supernatants due to a different pH in the LC–MS mobile phase, which contained 0.1% formic acid (pH ~3).

To test for potential cellular or extracellular enzymes that could lead to DFOB degradation, sterile spent medium and crude enzyme cell extracts from *P. biseptata* mycelium were prepared and incubated with DFOB for 72 h but no degradation of DFOB was detected in these experiments by LC–MS (data not shown). It was then tested if the presence of iron affected DFOB degradation by *P. biseptata* as suggested by the initial experiments (see above). It was found that when *P. biseptata* was incubated with FeDFOB in Fe replete MSB medium, there was no significant degradation (Figure 3B).

### *Degradation of protochelin by* P. biseptata

**FIGURE 4:**
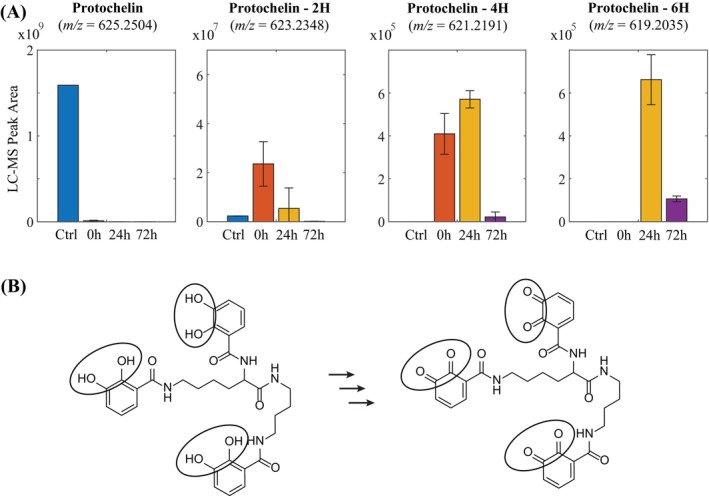
Removal of protochelin from supernatant solutions by *Pyrenophora biseptata* and detected degradation products (A). Iron‐chelating catechol moieties in protochelin and the quinone moieties in the degradation products are circled. Error bars indicate standard deviations from triplicate biological incubations. Structures of the protochelin degradation products were derived from tandem mass‐spectrometry fragmentation spectra (B).

Protochelin concentrations in solution decreased rapidly in incubations with *P. biseptata* mycelium (Figure 4A): in the first timepoint, taken immediately after inoculation, ~1% of the initial protochelin concentration was detected in the spent medium solution and after 24 and 72 h incubation, less than 0.1% was detected. The main detected protochelin degradation products indicated sum formulas equal to protochelin‐2H, and additional minor degradation products, including protochelin‐4H and protochelin‐6H. Analysis of MS/MS spectra showed that these compounds consisted of the protochelin structure with one, two or three catechol groups oxidized to quinones (Figure 4B). Extracted ion chromatograms of the main degradation product with *m/z* = 623.2348 (protochelin‐2H) showed three distinct peaks (retention times: 4.15, 4.23 and 4.39 min) with about equal intensities indicating any one of the three catechol groups in protochelin was oxidized leading to three different degradation products. As in the case of DFOB, the degradation of protochelin involved the iron‐chelating moieties in the siderophore, leading to a loss of the ability of the siderophore to bind iron. Peak areas of protochelin degradation products were around 1% of the protochelin standard in the first time point and less than 0.1% in the 24 and 72 h timepoints suggesting that the majority of protochelin removal from the solution was not accounted for by these degradation products. This agreed with the analysis of LC–UV–vis data, which showed only minor new UV–vis active catechol or quinone degradation products but not to the extent that these degradation products could account for a significant fraction of protochelin removed from the solution. This suggested that the bulk protochelin may have adsorbed onto cell surfaces. It is also possible that additional degradation products were not detected because they may have consisted of hydrophobic compounds that did not elute from the column.

### Siderophore production and degradation

**FIGURE 5:**
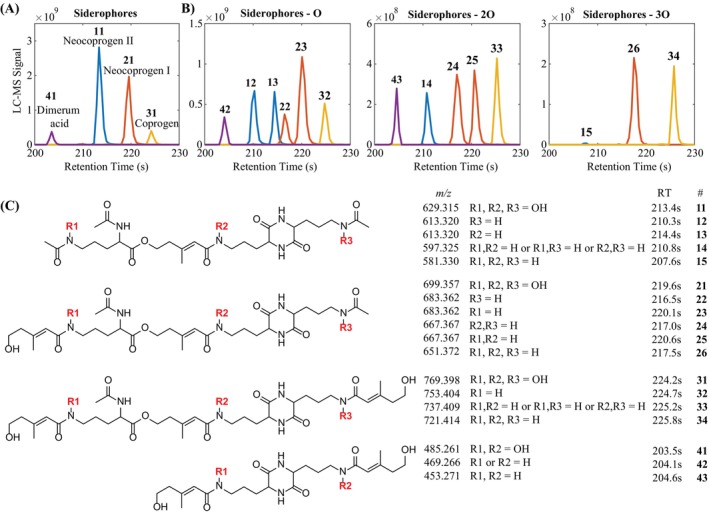
Siderophores produced by *Pyrenophora biseptata* (A) and related structures which had sum formulas with one, two or three oxygen less (B). Analysis of MS/MS spectra (Figure S3, Tables S3–S8) showed that the structures of these related compounds were identical to those of the four main siderophores with one, two or three hydroxamate groups reduced to amides, similar to the observed DFOB degradation (C). DFOB, desferrioxamine B; MS/MS, tandem mass spectrometry.

Beyond these degradation studies with the three siderophore models, we investigated which siderophores the tested fungi produce and whether they may undergo degradation by fungal activity similar to the siderophore standards. Siderophore assays (CAS assays) with iron‐limited MSB medium showed that of the four fungi, only *P. biseptata* was CAS positive. Following the initial CAS results, LC–MS/MS analyses were conducted to discover siderophores after 72 h incubation with *P. biseptata* mycelium in iron‐limited and iron‐replete media. Siderophore production was detected in iron‐limited medium but suppressed or not detected in iron‐replete medium. The main detected siderophores were matching in mass and MS/MS fragmentation to the known fungal siderophores neocoprogen I, neocoprogen II (Hossain et al., 1987), coprogen (Mawji et al., 2008) and dimerum acid (Bertrand et al., 2009; Lehner et al., 2013) (Figure 5A, Figure S2A–D, Table S3). Minor peaks were detected for several additional, structurally closely related, siderophores (Table S3). All detected siderophores from *P. biseptata* were in the coprogen family of structures. Coprogens are linear fungal trishydroxamate siderophores with the same iron‐chelating moieties as those in DFOB. Interestingly, as for DFOB, putative degradation products were detected with sum formulas less than one, two or three oxygen atoms (Figure 5B). MS/MS spectra showed that these structures were identical to those of the four main siderophores with one, two or three hydroxamate groups reduced to amides, analogous to the observed DFOB degradation (Figure 5C, Figure S3A–D, Tables S4–S8).

## DISCUSSION

In this study, we investigated the degradation of three representative siderophore structures by mycelium of ecologically and taxonomically diverse species of fungi found associated with plants and soil. The four isolates were selected because they are known to be prolific producers of extracellular enzymes for the acquisition of carbon (*P. chrysosporium* RP‐78 and BKM‐1767) (Haapala & Linko, 1993; Ray et al., 2012) or because they can colonize plants and can affect plant growth as in the case of *P. biseptata* (Peraza‐Jiménez et al., 2021) and *L. elongata* (Becker & Cubeta, 2020; Uehling et al., 2017). We found that *P. biseptata* incubations in iron‐limited MSB medium were associated with a significant decrease in dissolved concentrations of DFOB, protochelin, and, to a lesser degree, PDMA. *P. biseptata* also produced its own coprogen‐type siderophores in iron‐limited MSB medium. This suggested a notable biology of *P. biseptata* surrounding siderophore degradation, production and Fe acquisition. A closer investigation of the activity of *P. biseptata s*howed that the decrease in dissolved DFOB concentration was linked to the formation of DFOB degradation products in which one, two or three of the iron‐chelating hydroxamate groups were reduced to amides. No indication of enzymatic degradation was found during incubations of DFOB with cell lysates or sterile supernatant medium and a multi‐step mechanism might have been involved in the degradation in which fungal cells needed to remain intact. No degradation was observed with FeDFOB in iron‐replete medium, suggesting that either iron chelation protected the hydroxamate groups or that the biochemical mechanisms leading to DFOB degradation were not active under iron‐replete conditions. We also found that *P. biseptata* produced coprogen‐type siderophores, primarily neocoprogen I and neocoprogen II. Coprogens share similarities with DFOB in that they are linear trishydroxamate siderophores and *P. biseptata* degraded the siderophores it produced like it degraded DFOB. The reduction of tris‐hydroxamate siderophores by *P. biseptata* can be expected to have important implications for iron availability to plants. It has been shown via plant ^55^Fe radioisotope tracer short‐term uptake experiments that DFOB was not available to maize and oat plants (Bar‐Ness et al., 1992). In contrast, mono‐ and bis‐hydroxamates are plant‐available because of their reduced strength and lower reduction potential which enable plant uptake via ligand exchange or reductive iron uptake (Hördt et al., 2000; Winkelmann, 2007).

A few previous studies demonstrated that bacteria were able to degrade DFOB as part of an ability to grow on DFOB as the sole carbon source. DFOB degradation proceeded by hydrolysis of the amide bonds in DFOB via a serine protease in the nitrogen‐fixing bacterium *Mesorhizobium loti* (Zaya et al., 1998) or a specific DFOB hydrolase in multiple strains of nitrogen‐fixing bacteria *Azospirillum* (Winkelmann et al., 1999) but the hydroxamate groups remained intact. Another study showed that the tris hydroxamate ferrichrome A was utilized by the soil bacterium *Pseudomonas* sp. isolate FC1 as a carbon source and involved degradation via hydrolysis of the peptide bonds to yield simpler hydroxamate structures (Warren & Neilands, 1964). The presence of Fe and the formation of Fe‐siderophore chelates protected siderophores from the degradation reaction. A separate mechanism was involved in several studies which have shown the enzymatic degradation of iron‐fusarinine complexes as part of a cellular mechanism to recover iron from these siderophores in the cytosol or the periplasm (Ecker et al., 2018; Gründlinger et al., 2013; Kragl et al., 2007). The degradation involved fungal esterases that hydrolyze the cyclic tris‐hydroxamate fusarinine C at the ester bonds to form mono‐ and bis‐hydroxamate products. The reaction products had lower iron affinities, which promoted cellular iron utilization (Kragl et al., 2007).

In contrast to our study, these previous reports showed hydrolysis of the amide bonds or ester bonds in siderophores while the hydroxamate groups remained intact. To our knowledge, siderophore degradation via microbial reduction of hydroxamate groups to amides has not been reported previously and may have important implications for metal uptake and the fate of siderophores in soil environments. Because of the potential implications, we provide some preliminary discussion exploring putative mechanisms for observed changes in dissolved siderophore concentration and structure. However, we specifically note that definitively establishing the mechanism of DFOB degradation was outside the scope of this work and is the subject of ongoing studies in this laboratory.

An abiotic auto‐decomposition reaction of Fe^II^‐DFOB complexes was previously observed under anaerobic conditions in which the oxidation of two Fe^II^ ions was coupled to the reduction of a hydroxamate group to an amide (Equation 1) (Farkas et al., 2001; Kim et al., 2009).

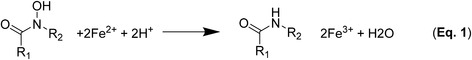

The initial reaction rate was rapid under neutral to acidic pH values, such as those found in the fungal incubations in our study (pH = 4.5). A mechanism as described in Equation (1) could explain the observed DFOB and coprogen siderophore degradation if Fe^II^ was generated in our aerobic experiments. Under aerobic conditions, DFOB was present as free DFOB or as Fe^III^‐DFOB chelate. Generally, fungi can acquire iron from siderophores via (1) a non‐reductive system that utilizes Fe^III^‐siderophore transporters or ligand‐exchange of Fe^III^ at the cell surface, and (2) by reduction of the Fe^III^‐siderophore complex to a weaker and more labile Fe^II^ complex at the plasma membrane followed by Fe^II^ exchange with permeases or oxidases for cellular uptake (Pecoraro et al., 2021; Philpott, 2006; Winkelmann, 2007). Iron acquisition mechanisms by fungi have been elucidated in detail with the *Saccharomyces cerevisiae* model. *S. cerevisiae* does not produce siderophores, but is capable of acquiring Fe from siderophores via non‐reductive and reductive uptake pathways (Yun et al., 2000). The reductive system involves plasma membrane‐bound ferric reductases (FREs) that are produced under conditions of iron (and, in some cases, copper) limitation, primarily Fre1p and Fre2p. Fe reduction is followed by either transport of Fe^II^ via a high‐affinity ferrous transporter or re‐oxidation of Fe^II^ to Fe^III^ via a multicopper ferroxidase (Fet3) and transport via a permease (Ftr1) (Philpott, 2006; Yun et al., 2000). A broad substrate specificity of Fre1p and Fre2p enables *S. cerevisiae* to acquire iron from a wide range of sources, including iron bound to hydroxamate siderophores, such as DFOB and catechol siderophores, such as enterobactin (Philpott, 2006; Yun et al., 2000; Yun et al., 2001). The reduction of stable and inert Fe^III^–tris hydroxamate siderophore chelates by physiologic reductants such as NADPH becomes possible by coupling the reduction with chelation of the Fe^II^ ion in a high‐affinity transporter, combined with a hydrophobic environment and reduced pH at the cell surface (Dhungana & Crumbliss, 2005; Philpott, 2006). Iron reduction thus enables ligand exchange both thermodynamically and kinetically to access siderophore‐bound iron (Dhungana & Crumbliss, 2005). To get preliminary evidence whether fungal reductive iron acquisition led to DFOB degradation, we cultured *S. cerevisiae* S288C under the same conditions as *P. biseptata*. LC–MS analysis of Fe‐limited *S. cerevisiae* S288C supernatants after 72 h of incubation indeed showed significant formation of the DFOB‐O degradation product, although compared to the *P. biseptata* incubations, only a minor fraction of DFOB had reacted (~4%) (Figure S4). The DFOB‐2O and DFOB‐3O degradation products were not detected, likely because they were below the detection limit. Orthologs of the *S. cerevisiae* reductive iron uptake system are present in other fungi and possibly widely distributed among fungi (Kosman, 2003): a *FreB* metalloreductase is involved in the *Aspergillus fumigatus* iron starvation response (Blatzer et al., 2011) and the black fungus pathogen *Cladophialophora carrionii* has been found to secrete the extracellular ferricrocin while also expressing reductive iron uptake genes similar to *S. cerevisiae* (Bailão et al., 2023). As pointed out by Bailão et al. (2023), the oxidase‐permease components of the reductive iron uptake system of *S. cerevisiae* are also conserved in other human pathogens, including *Candida albicans* (Ramanan & Wang, 2000), *Cryptococcus neoformans* (Jung et al., 2008), and in the plant pathogens *Ustilago maydis* (Eichhorn et al., 2006) and *Fusarium graminearum* (Park et al., 2007). There is no available whole‐genome sequence of *P. biseptata* but the wide distribution of high‐affinity iron‐reductive‐uptake systems in fungi supports the hypothesis that our mechanism may involve the formation of ferrous iron species. If the discussed mechanistic link between high‐affinity reductive iron uptake and degradation of hydroxamate siderophores is correct, any fungal plant symbionts that utilize the high‐affinity reductive iron‐uptake system in the presence of tris‐hydroxamate siderophores may be expected to enhance iron‐bioavailability to plants by forming bis‐ and mono‐hydroxamates in competitive low‐iron environments.

Taken together, reductive iron acquisition was a plausible explanation for the observed degradation reaction in our study. We hypothesized that reductive iron uptake activity by *P. biseptata* involved the formation of transient Fe^II^‐DFOB or Fe^II^‐coprogen, followed by a re‐oxidation of Fe^II^ to Fe^III^ and reduction of a siderophore hydroxamate group. If such a mechanism was involved in DFOB degradation, we need to be able to explain the observation of DFOB degradation in Fe limited medium whereas FeDFOB complexes were not degraded in Fe replete medium. Fe‐limited media are not completely iron‐free but contain iron as trace‐contaminants enabling significant growth of fungal cultures, which can explain degradation in Fe‐limited media. Conversely, Fe‐replete media are not expected to result in the degradation of FeDFOB because of suppression of the FRE ferric reductases and high‐affinity ferrous iron transporters involved in Fe acquisition from FeDFOB in Fe‐replete conditions (Philpott, 2006). Supporting a suppression of iron starvation response in Fe‐replete medium, *P. biseptata* did not produce coprogen siderophores.

The specific activity to degrade as well as produce tris‐hydroxamate siderophores by *P. biseptata* suggested a biological role linked to the efficient uptake of iron. The reduction of the iron‐chelating hydroxamate groups (Equation 1) leads to a progressive loss of Fe chelating ability, enhancing reactivity for further Fe reduction or ligand exchange, but comes at the cost of the fungal siderophore being degraded. Therefore, this activity may be beneficial in the presence of high siderophore concentrations, for example, in the presence of siderophores from competing organisms. Supporting this idea, the reductive iron uptake from tris‐hydroxamate siderophores has been reported to be preferred at high siderophore concentrations, while non‐reductive uptake mechanisms are prevalent at low siderophore concentrations (Lesuisse et al., 2001; Philpott, 2006). Nearly all fungi are capable of taking up Fe via the non‐reductive uptake system that is specific for iron‐siderophore chelates (Hsiang & Baillie, 2005; Philpott, 2006). It can be hypothesized that *P. biseptata* adapted to growing in both an environment with low iron‐chelator concentrations and low iron availability by producing its own siderophores, and taking up iron in an environment of high siderophore concentration from the activity of competing bacteria and fungi using a reductive uptake system.

Aside from DFOB, *P. biseptata* also rapidly removed protochelin from the solution. Minor degradation peaks could be identified and showed oxidation of the 2,3‐dihydroxybenzoate groups to quinone groups. The oxidation of quinones in protochelin was previously observed by cyclic voltammetry and interpreted as a reversible, redox‐mediated method to release Fe from the Fe^III^‐protochelin complexes because the quinone groups have greatly reduced Fe^III^ binding affinities (Harrington et al., 2012). Most of the protochelin removed from the solution was not accounted for by potential degradation products in our study, either because it was taken up and metabolized internally by the fungus, or because it was adsorbed, or degradation products evaded detection. Experiments to recover protochelin by cell extraction with methanol were not successful, implying potential degradation following adsorption of protochelin on the surface of fungal cells. Our previous studies with protochelin have shown a propensity of the hydrophobic protochelin to adsorb to particles and colloids in soil extract solutions (Rai et al., 2020) and the catechol groups can also react in oxidative polymerization processes forming hydrophobic products (Baars et al., 2015; Pinnataip & Lee, 2021). The rapid removal of the bulk of protochelin within minutes after addition to the fungal mycelium without the observation of corresponding degradation products in solution suggested that protochelin or protochelin degradation products may have adsorbed on cellular surfaces. Given the strong removal of DFOB and protochelin by *P. bispetata* in contrast to the other three tested fungi, it appears that the observed oxidation of protochelin may have involved reductive iron uptake at the *P. biseptata* plasma membrane similar to that discussed above for DFOB. Reductive iron uptake from chelates with the tris‐catechol siderophore enterobactin has been reported previously with *S. cerevisae* and involved the same ferric reductases that can also reduce Fe^III^ bound to DFOB (FRE1, FRE2) (Yun et al., 2001). In the presence of oxygen, high iron redox activity at the cell surface may have resulted in the rapid formation of polymeric protochelin oxidation products.

Common to the observed DFOB and protochelin degradation in our study was that the reaction occurred specifically in the iron‐chelating groups so siderophores were compromised in their Fe‐binding ability. The impact is that iron becomes more available to plants and microbes that are not able to access iron‐siderophore chelates. The observed siderophore degradation by *P. biseptata* may be a widespread ability among fungi and we hypothesize that degradation involves fungal reductive iron acquisition systems. Future studies will need to test and mechanistically establish this hypothesized link between high‐affinity reductive iron uptake and siderophore degradation as well as consequences for iron availability to plants.

## AUTHOR CONTRIBUTIONS

**Katie S. French:** Investigation (lead); methodology (equal); writing – original draft (supporting); writing – review and editing (supporting). **Emmanuel Chukwuma:** Investigation (equal); methodology (equal); supervision (supporting); validation (equal); writing – original draft (supporting); writing – review and editing (supporting). **Ilan Linshitz:** Investigation (equal); methodology (equal); validation (equal); writing – original draft (supporting). **Kosuke Namba:** Resources (equal); writing – review and editing (supporting). **Marc A. Cubeta:** Conceptualization (supporting); methodology (supporting); resources (equal); supervision (equal); writing – original draft (supporting); writing – review and editing (supporting). **Owen W. Duckworth:** Conceptualization (equal); funding acquisition (lead); project administration (equal); resources (equal); supervision (supporting); writing – original draft (equal); writing – review and editing (equal). **Oliver Baars:** Conceptualization (lead); data curation (lead); formal analysis (lead); funding acquisition (equal); investigation (equal); methodology (equal); project administration (lead); resources (equal); software (equal); supervision (lead); validation (lead); visualization (lead); writing – original draft (lead); writing – review and editing (lead).

## CONFLICT OF INTEREST STATEMENT

The authors declare no conflicts of interest.

## Supporting information


**DATA S1.** Supporting Information.

## Data Availability

LC–MS/MS raw data for review are available at ftp://massive.ucsd.edu/v02/msv000092898 and https://doi.org/10.25345/C5DZ03C02
