# Supplementary material for: Inactivation of siderophore iron‐chelating moieties by the fungal wheat root symbiont Pyrenophora biseptata
Source: Environ Microbiol Rep. 2024 Jan 19;16(1):e13234. doi: 10.1111/1758-2229.13234 (PMC10866069; doi:10.1111/1758-2229.13234)
Supplement: Supplementary file 1 — DATA S1. Supporting Information. [file EMI4-16-e13234-s001.docx]

**Supplemental Information**

**Table S1:** Siderophore standards, ionization mode, and *m/z* values used in LC-MS analysis with Single-Ion-Monitoring (see methods).

| **Siderophore** | **Ionization** | **Exact mass (M)** | ***m/z* (MH^+^)** | ***m/z* (M-H)^-^** | ***z*** |
| --- | --- | --- | --- | --- | --- |
| DFOB | Positive | 560.3534 | 561.4 |  | 1 |
| DFOB – O | Positive | 544.3585 | 545.4 |  | 1 |
| DFOB – 2O | Positive | 528.3635 | 529.4 |  | 1 |
| FeDFOB | Positive | 613.2648 | 614.3 |  | 1 |
| PDMA | Positive | 318.1427 | 319.1 |  | 1 |
| FePDMA | Positive | 371.0542 | 372.1 |  | 1 |
| Protochelin | Negative | 624.2431 |  | 623.2 | 1 |
| Protochelin – 2H | Negative | 622.2256 |  | 621.2 | 1 |
| FeProtochelin | Negative | 677.1546 |  | 676.2 | 1 |

**Table S2:** MS/MS fragmentation of DFOB and DFOB reaction products referenced in Fig. 3

| **DFOB** | |  | **DFOB-O** | |  | **DFOB-2O** | |  | **DFOB-3O** | |
| --- | --- | --- | --- | --- | --- | --- | --- | --- | --- | --- |
| Precursor *m/z* | 561.3594 |  | Precursor *m/z* | 545.3646 |  | Precursor *m/z* | 529.3697 |  | Precursor *m/z* | 513.3747 |
| Fragment *m/z* | Intensity (%) |  | Fragment *m/z* | Intensity (%) |  | Fragment *m/z* | Intensity (%) |  | Fragment *m/z* | Intensity (%) |
| 401.2396 | 3.0 |  | 545.365 | 2.7 |  | 529.3697 | 3.2 |  | 532.9739 | 1.0 |
| 361.2444 | 2.3 |  | 385.2445 | 2.0 |  | 411.26 | 1.4 |  | 513.3757 | 12.4 |
| 319.2336 | 10.7 |  | 361.2442 | 2.1 |  | 385.2446 | 2.4 |  | 495.3647 | 4.9 |
| 283.1288 | 2.1 |  | 345.2495 | 1.9 |  | 345.2493 | 6.0 |  | 472.8612 | 1.1 |
| 243.1337 | 51.5 |  | 319.2341 | 8.5 |  | 303.2388 | 45.9 |  | 411.26 | 18.6 |
| 201.1231 | 100.0 |  | 303.2389 | 25.9 |  | 267.1335 | 5.0 |  | 393.2501 | 1.4 |
| 183.1125 | 5.3 |  | 283.1291 | 2.5 |  | 227.1388 | 100.0 |  | 369.2492 | 39.4 |
| 168.1017 | 1.4 |  | 267.134 | 1.8 |  | 201.1231 | 17.6 |  | 351.2387 | 22.6 |
| 166.086 | 1.2 |  | 243.1337 | 18.8 |  | 186.1121 | 5.0 |  | 329.2544 | 35.3 |
| 165.1021 | 7.7 |  | 228.1421 | 1.4 |  | 185.1282 | 72.0 |  | 329.2289 | 1.1 |
| 154.086 | 6.6 |  | 227.1389 | 100.0 |  | 183.1126 | 2.7 |  | 312.2276 | 2.5 |
| 144.1017 | 19.1 |  | 201.1232 | 63.6 |  | 168.1017 | 21.0 |  | 311.2442 | 2.2 |
| 140.0703 | 1.7 |  | 186.1123 | 5.3 |  | 167.1176 | 2.2 |  | 287.2438 | 32.5 |
| 138.0911 | 2.2 |  | 185.1283 | 47.0 |  | 165.1019 | 2.0 |  | 285.1446 | 1.5 |
| 119.1179 | 3.5 |  | 184.0967 | 1.6 |  | 154.0858 | 1.2 |  | 270.218 | 3.2 |
| 112.039 | 1.5 |  | 183.1127 | 4.6 |  | 145.1334 | 1.6 |  | 269.2337 | 2.9 |
| 102.0912 | 17.4 |  | 168.1017 | 12.4 |  | 128.1068 | 43.7 |  | 267.1336 | 33.3 |
| 100.0392 | 5.1 |  | 167.118 | 1.2 |  | 119.1179 | 1.6 |  | 266.1494 | 7.0 |
| 88.0392 | 1.2 |  | 165.1021 | 6.3 |  | 103.1229 | 6.1 |  | 241.2673 | 1.1 |
|  |  |  | 154.0862 | 4.6 |  | 102.0913 | 2.9 |  | 227.1388 | 100.0 |
|  |  |  | 144.1018 | 7.2 |  | 100.0392 | 5.7 |  | 226.1544 | 2.0 |
|  |  |  | 138.0913 | 1.5 |  | 86.0963 | 21.7 |  | 203.139 | 1.1 |
|  |  |  | 128.1069 | 35.5 |  | 84.0806 | 12.6 |  | 186.1121 | 6.2 |
|  |  |  | 119.1182 | 3.8 |  | 72.0443 | 3.6 |  | 185.1282 | 86.1 |
|  |  |  | 103.123 | 3.4 |  |  |  |  | 184.1441 | 4.1 |
|  |  |  | 102.0913 | 10.8 |  |  |  |  | 168.1017 | 61.3 |
|  |  |  | 100.0392 | 6.4 |  |  |  |  | 167.1176 | 27.4 |
|  |  |  | 86.0964 | 10.8 |  |  |  |  | 145.1334 | 28.3 |
|  |  |  | 84.0807 | 19.3 |  |  |  |  | 128.1068 | 33.4 |
|  |  |  | 165.102 | 10.1 |  |  |  |  | 110.745 | 1.1 |
|  |  |  | 161.1282 | 16.2 |  |  |  |  | 103.1228 | 6.7 |
|  |  |  | 154.086 | 5.3 |  |  |  |  | 100.0392 | 9.1 |
|  |  |  | 144.1018 | 20.7 |  |  |  |  | 86.0963 | 42.8 |
|  |  |  | 140.0708 | 1.5 |  |  |  |  | 84.3068 | 1.1 |
|  |  |  | 138.091 | 2.8 |  |  |  |  | 81.9933 | 1.0 |
|  |  |  | 131.0812 | 1.5 |  |  |  |  | 72.0443 | 2.4 |
|  |  |  | 128.1069 | 17.3 |  |  |  |  | 69.9539 | 1.2 |
|  |  |  | 119.1179 | 5.3 |  |  |  |  |  |  |
|  |  |  | 113.0599 | 1.5 |  |  |  |  |  |  |
|  |  |  | 112.0391 | 2.5 |  |  |  |  |  |  |
|  |  |  | 103.1229 | 2.0 |  |  |  |  |  |  |
|  |  |  | 102.0913 | 20.4 |  |  |  |  |  |  |
|  |  |  | 100.0392 | 17.8 |  |  |  |  |  |  |
|  |  |  | 98.0965 | 1.2 |  |  |  |  |  |  |
|  |  |  | 86.0963 | 16.6 |  |  |  |  |  |  |
|  |  |  | 84.0807 | 45.5 |  |  |  |  |  |  |
|  |  |  | 72.0442 | 2.5 |  |  |  |  |  |  |
|  |  |  | 69.0698 | 2.5 |  |  |  |  |  |  |

**Table S3:** Siderophores produced by *P. biseptata* in iron-limited culture supernatants (see methods). Characteristic MS/MS fragments shown in Figure S2 are shown. MS/MS spectra for Neocoprogen I and Coprogen matched GNPS library reference spectra.

| ***m/z* exp** | ***m/z* calc** | **Δ ppm** | **RT (min)** | **Peak Area** | **Sum formula** | **ID** | **Characteristic MS/MS fragments** | **GNPS Reference Spectrum** |
| --- | --- | --- | --- | --- | --- | --- | --- | --- |
| 629.3146 | 629.3130 | 2.7 | 3.56 | 6.3E+09 | C27H44N6O11 | Neocoprogen II | 173.0919, 128.0706, 303.1667 |  |
| 699.3565 | 699.3551 | 2.1 | 3.66 | 4.7E+09 | C31H50N6O12 | Neocoprogen I | 113.0603, 173.0919, 128.0706, 303.1667 | CCMSLIB00001059086 |
| 769.3984 | 769.3966 | 2.4 | 3.74 | 8.8E+08 | C35H56N6O13 | Coprogen | 113.0603, 173.0919, 397.1975, 355.1976, 243.1335 | CCMSLIB00001059084 |
| 485.2611 | 485.2597 | 3.1 | 3.39 | 7.1E+08 | C22H36N4O8 | Dimerum acid | 113.0603, 243.1335 |  |
| 657.3459 | 657.3443 | 2.6 | 3.33 | 6.5E+08 | C29H48N6O11 | Neocoprogen I - H2C2O2 | 113.0603, 173.0916, 128.0706, 303.1658, 243.1335 |  |
| 601.3197 | 601.3182 | 2.6 | 3.25 | 6.2E+08 | C26H44N6O10 | Neocoprogen I - C5H6O2 | 113.0603, 128.0706, 303.1658, 141.1022, 205.1182, 159.1127 |  |
| 587.3041 | 587.3025 | 2.8 | 3.23 | 4.5E+08 | C25H42N6O10 | Neocoprogen II - H2C2O2 | 173.0919, 128.0706 |  |
| 727.3878 | 727.3860 | 2.5 | 3.41 | 9.5E+07 | C33H54N6O12 | Coprogen B | no MS/MS spectrum collected |  |
| 715.3514 | 715.3494 | 2.9 | 3.49 | 4.3E+07 | C31H50N6O13 | Hydroxy-neocoprogen I | no MS/MS spectrum collected |  |

**Table S4:** MS/MS fragments (illustrated in Figure S3) used to assign the coprogen degradation products in Figure 5.

| **Peak No (see Fig. 5)** | **Characteristic MS/MS fragments** |
| --- | --- |
| **12** | 112.0757, 70.0650, 287.1718 (only one of the right two hydroxamate groups is reduced) |
| **13** | 128.0706, 86.0599, 287.1718 |
| **14** | 112.0757, 128.0706, 70.0650, 86.0599, 271.1769, 287.1718 |
| **15** | no MS/MS spectrum available |
| **22** | 112.0757, 70.0650, 287.1718 |
| **23** | 115.0865, 303.1667 |
| **24** | 271.1769 |
| **25** | 287.1718, 115.0865 |
| **26** | 112.0757, 115.0865, 70.0650, 271.1769 |
| **32** | 381.2026 |
| **33** | 381.2026, 227.1386, 339.2027, 323.2079 |
| **34** | 381.2026, 227.1386, 323.2078 |

**Table S5:** MS/MS fragmentation spectra used to assign Neocoprogen II and corresponding degradation products in Figure 5.

| **#11** | |  | **#12** | |  | **#13** | |  | **#14** | |
| --- | --- | --- | --- | --- | --- | --- | --- | --- | --- | --- |
| Precursor *m/z* | 629.3129 |  | Precursor *m/z* | 613.3179 |  | Precursor *m/z* | 613.3182 |  | Precursor *m/z* | 597.3230 |
| Fragment *m/z* | Intensity (%) |  | Fragment *m/z* | Intensity (%) |  | Fragment *m/z* | Intensity (%) |  | Fragment *m/z* | Intensity (%) |
| 472.8634 | 0.4 |  | 614.2722 | 1.3 |  | 614.2715 | 0.6 |  | 555.3135 | 3.5 |
| 411.5243 | 0.3 |  | 535.7814 | 0.3 |  | 613.3196 | 0.5 |  | 537.3024 | 1.7 |
| 303.3865 | 0.3 |  | 496.1628 | 1.0 |  | 571.3079 | 2.2 |  | 525.8546 | 0.6 |
| 303.1662 | 0.7 |  | 474.3988 | 0.3 |  | 553.2975 | 1.8 |  | 478.2647 | 0.8 |
| 288.4533 | 0.3 |  | 472.8594 | 0.6 |  | 529.2972 | 0.4 |  | 477.7274 | 0.6 |
| 285.1565 | 2.7 |  | 468.1650 | 0.7 |  | 496.2737 | 0.3 |  | 472.7739 | 0.9 |
| 284.1575 | 0.3 |  | 414.1564 | 1.3 |  | 496.1609 | 0.6 |  | 365.2179 | 2.1 |
| 270.1439 | 0.4 |  | 397.1292 | 0.6 |  | 474.4108 | 0.3 |  | 297.1554 | 1.8 |
| 266.7634 | 0.3 |  | 381.2143 | 0.4 |  | 472.8684 | 0.4 |  | 287.1709 | 3.1 |
| 257.7534 | 0.3 |  | 315.0631 | 0.7 |  | 468.1648 | 0.7 |  | 271.1765 | 12.5 |
| 241.2520 | 0.4 |  | 307.4664 | 0.3 |  | 429.4384 | 0.3 |  | 271.1400 | 0.7 |
| 233.1131 | 0.4 |  | 299.0689 | 0.5 |  | 414.1550 | 1.0 |  | 270.1448 | 7.8 |
| 226.1184 | 3.6 |  | 287.1705 | 1.9 |  | 399.2225 | 0.6 |  | 269.1608 | 2.3 |
| 215.1028 | 0.5 |  | 285.1555 | 1.2 |  | 397.1292 | 0.5 |  | 261.9319 | 0.6 |
| 210.1224 | 0.5 |  | 270.1463 | 0.3 |  | 381.2134 | 2.6 |  | 254.1499 | 23.2 |
| 208.1079 | 0.5 |  | 269.1608 | 3.1 |  | 342.8703 | 0.3 |  | 253.1661 | 7.9 |
| 195.1122 | 0.5 |  | 255.0788 | 0.3 |  | 339.2024 | 1.2 |  | 241.2070 | 0.7 |
| 193.0969 | 1.1 |  | 254.1499 | 1.1 |  | 321.1930 | 0.8 |  | 238.1180 | 0.7 |
| 191.1027 | 0.4 |  | 253.0636 | 0.4 |  | 315.0637 | 0.7 |  | 236.1397 | 1.0 |
| 187.1074 | 0.9 |  | 252.1342 | 0.5 |  | 313.1496 | 0.5 |  | 228.1343 | 2.2 |
| 184.7034 | 0.3 |  | 234.8669 | 0.3 |  | 312.1546 | 0.3 |  | 217.1183 | 1.1 |
| 183.4151 | 0.3 |  | 226.1182 | 2.5 |  | 287.1713 | 6.7 |  | 212.1394 | 2.1 |
| 174.0952 | 0.8 |  | 217.1179 | 0.4 |  | 285.1433 | 0.5 |  | 210.1234 | 1.6 |
| 173.0918 | 100.0 |  | 210.1234 | 0.6 |  | 271.1397 | 0.6 |  | 203.6904 | 0.6 |
| 162.2937 | 0.3 |  | 208.1073 | 0.4 |  | 270.1446 | 12.7 |  | 199.1079 | 11.0 |
| 155.0813 | 7.2 |  | 200.0367 | 0.3 |  | 269.1608 | 4.1 |  | 195.1127 | 9.3 |
| 146.3174 | 0.3 |  | 199.1077 | 9.8 |  | 253.1295 | 0.6 |  | 194.1288 | 1.6 |
| 145.0970 | 2.2 |  | 199.0961 | 0.6 |  | 252.1337 | 0.4 |  | 192.1133 | 1.2 |
| 140.0734 | 0.3 |  | 195.1123 | 0.6 |  | 245.1609 | 0.4 |  | 189.3848 | 0.6 |
| 131.0814 | 39.0 |  | 193.0972 | 2.0 |  | 241.2575 | 0.3 |  | 187.1079 | 0.7 |
| 128.0705 | 11.0 |  | 192.1133 | 0.8 |  | 238.1179 | 0.8 |  | 184.6788 | 0.9 |
| 127.0865 | 1.2 |  | 187.1076 | 0.5 |  | 233.1131 | 0.7 |  | 173.0920 | 80.9 |
| 114.0548 | 10.9 |  | 184.7058 | 0.5 |  | 228.1340 | 3.2 |  | 171.1129 | 4.6 |
| 113.0707 | 6.7 |  | 181.0972 | 0.4 |  | 227.1758 | 0.3 |  | 163.5618 | 0.6 |
| 113.0596 | 1.2 |  | 175.1074 | 0.5 |  | 227.1500 | 0.4 |  | 157.0970 | 25.3 |
| 112.0758 | 0.3 |  | 174.0951 | 0.4 |  | 215.1024 | 1.0 |  | 155.0816 | 6.3 |
| 97.6973 | 0.3 |  | 173.0919 | 49.5 |  | 211.1077 | 0.5 |  | 145.0972 | 1.1 |
| 95.0526 | 0.5 |  | 171.1127 | 4.3 |  | 210.1487 | 0.8 |  | 140.8370 | 0.6 |
| 95.0490 | 5.3 |  | 158.0812 | 0.6 |  | 210.1234 | 4.2 |  | 139.0866 | 2.4 |
| 90.2185 | 0.3 |  | 157.0970 | 23.7 |  | 195.1127 | 5.1 |  | 137.7252 | 0.6 |
| 86.0599 | 22.5 |  | 155.0814 | 3.8 |  | 194.1288 | 0.4 |  | 131.0815 | 41.4 |
| 85.0759 | 0.6 |  | 149.8852 | 0.2 |  | 193.0968 | 1.0 |  | 129.1022 | 2.3 |
| 82.0943 | 0.4 |  | 149.5689 | 0.3 |  | 192.1126 | 1.4 |  | 129.0899 | 0.5 |
| 70.2552 | 0.3 |  | 145.0971 | 0.7 |  | 191.1025 | 0.5 |  | 128.0705 | 10.4 |
| 70.0650 | 1.6 |  | 139.1606 | 0.3 |  | 187.1073 | 0.7 |  | 115.0866 | 8.5 |
| 68.0495 | 0.5 |  | 139.0865 | 2.3 |  | 185.3105 | 0.3 |  | 114.0549 | 8.5 |
| 67.0541 | 1.2 |  | 138.6906 | 0.3 |  | 184.7046 | 0.3 |  | 113.0789 | 0.7 |
|  |  |  | 131.0814 | 21.4 |  | 183.5429 | 0.3 |  | 113.0710 | 7.2 |
|  |  |  | 129.1023 | 1.4 |  | 174.0958 | 0.8 |  | 113.0595 | 3.3 |
|  |  |  | 128.0706 | 5.6 |  | 173.0918 | 100.0 |  | 112.0757 | 100.0 |
|  |  |  | 127.0866 | 0.4 |  | 169.5284 | 0.3 |  | 95.0491 | 22.0 |
|  |  |  | 118.2161 | 0.3 |  | 167.0813 | 0.8 |  | 95.0452 | 0.8 |
|  |  |  | 115.0865 | 5.6 |  | 159.7301 | 0.3 |  | 86.0600 | 22.5 |
|  |  |  | 114.0548 | 6.2 |  | 156.0896 | 0.4 |  | 85.1724 | 0.7 |
|  |  |  | 113.0789 | 0.7 |  | 155.0813 | 7.6 |  | 85.0759 | 0.6 |
|  |  |  | 113.0709 | 3.8 |  | 145.9406 | 0.3 |  | 70.6444 | 0.5 |
|  |  |  | 113.0596 | 0.8 |  | 145.0970 | 2.6 |  | 70.0651 | 28.8 |
|  |  |  | 112.0756 | 100.0 |  | 140.0901 | 0.3 |  | 69.2583 | 0.6 |
|  |  |  | 106.3973 | 0.3 |  | 132.0848 | 0.4 |  | 67.8381 | 0.6 |
|  |  |  | 102.0547 | 0.3 |  | 131.0813 | 45.7 |  | 67.0542 | 5.6 |
|  |  |  | 95.0490 | 5.9 |  | 128.1139 | 0.3 |  |  |  |
|  |  |  | 94.2744 | 0.3 |  | 128.0705 | 14.1 |  |  |  |
|  |  |  | 89.3210 | 0.2 |  | 127.0865 | 1.6 |  |  |  |
|  |  |  | 86.0600 | 12.7 |  | 125.0706 | 0.3 |  |  |  |
|  |  |  | 84.0809 | 0.3 |  | 124.6813 | 0.3 |  |  |  |
|  |  |  | 82.0988 | 0.3 |  | 115.0865 | 4.5 |  |  |  |
|  |  |  | 70.0651 | 23.1 |  | 114.0548 | 9.0 |  |  |  |
|  |  |  | 69.9486 | 0.3 |  | 113.5952 | 0.3 |  |  |  |
|  |  |  | 67.4167 | 0.3 |  | 113.0708 | 8.4 |  |  |  |
|  |  |  | 67.0542 | 1.2 |  | 113.0596 | 2.8 |  |  |  |
|  |  |  |  |  |  | 112.0756 | 0.6 |  |  |  |
|  |  |  |  |  |  | 98.0599 | 0.4 |  |  |  |
|  |  |  |  |  |  | 97.6969 | 0.4 |  |  |  |
|  |  |  |  |  |  | 96.0443 | 0.8 |  |  |  |
|  |  |  |  |  |  | 95.0490 | 16.1 |  |  |  |
|  |  |  |  |  |  | 94.7586 | 0.3 |  |  |  |
|  |  |  |  |  |  | 90.6315 | 0.3 |  |  |  |
|  |  |  |  |  |  | 86.0599 | 26.9 |  |  |  |
|  |  |  |  |  |  | 85.0759 | 0.9 |  |  |  |
|  |  |  |  |  |  | 84.0804 | 0.3 |  |  |  |
|  |  |  |  |  |  | 83.9547 | 0.3 |  |  |  |
|  |  |  |  |  |  | 72.4551 | 0.3 |  |  |  |
|  |  |  |  |  |  | 70.0650 | 2.7 |  |  |  |
|  |  |  |  |  |  | 69.9493 | 0.3 |  |  |  |
|  |  |  |  |  |  | 69.0572 | 0.3 |  |  |  |
|  |  |  |  |  |  | 68.0495 | 0.7 |  |  |  |
|  |  |  |  |  |  | 67.0542 | 2.6 |  |  |  |

**Table S6:** MS/MS fragmentation spectra used to assign Neocoprogen I and corresponding degradation products in Figure 5.

| Precursor *m/z* | 699.3545 |  | Precursor *m/z* | 683.3602 |  | Precursor *m/z* | 683.3602 |  | Precursor *m/z* | 667.3651 |  | Precursor *m/z* | 667.3650 |  | Precursor *m/z* | 651.3699 |
| --- | --- | --- | --- | --- | --- | --- | --- | --- | --- | --- | --- | --- | --- | --- | --- | --- |
| Fragment *m/z* | Int (%) |  | Fragment *m/z* | Int (%) |  | Fragment *m/z* | Int (%) |  | Fragment *m/z* | Int (%) |  | Fragment *m/z* | Int (%) |  | Fragment *m/z* | Int (%) |
| 587.2983 | 0.7 |  | 582.2905 | 1.4 |  | 716.1094 | 0.5 |  | 555.3129 | 6.3 |  | 637.4042 | 0.8 |  | 641.989 | 0.6 |
| 474.3994 | 0.7 |  | 571.3077 | 1.4 |  | 666.4001 | 0.5 |  | 537.3016 | 2.1 |  | 625.3566 | 2.2 |  | 609.360 | 1.6 |
| 473.0003 | 0.8 |  | 526.8636 | 1.4 |  | 571.3079 | 4.9 |  | 495.2937 | 1.5 |  | 607.3447 | 2.6 |  | 591.350 | 2.3 |
| 397.1993 | 1.5 |  | 474.3975 | 1.7 |  | 553.2966 | 2.6 |  | 478.2677 | 3.6 |  | 555.3659 | 1.3 |  | 559.299 | 0.6 |
| 380.1930 | 0.6 |  | 452.2723 | 1.6 |  | 529.3004 | 0.9 |  | 463.0604 | 0.7 |  | 555.3135 | 17.5 |  | 540.322 | 0.8 |
| 303.1660 | 3.2 |  | 367.0295 | 4.2 |  | 511.2895 | 1.1 |  | 409.9456 | 0.7 |  | 538.2858 | 1.3 |  | 539.318 | 18.7 |
| 285.1572 | 4.9 |  | 359.0296 | 1.6 |  | 494.2608 | 1.0 |  | 383.2282 | 1.2 |  | 537.3026 | 6.6 |  | 522.292 | 0.9 |
| 281.5170 | 0.5 |  | 349.0199 | 12.4 |  | 493.2769 | 0.7 |  | 381.2021 | 7.1 |  | 517.0208 | 0.6 |  | 521.308 | 6.2 |
| 281.2018 | 0.6 |  | 348.0122 | 1.4 |  | 472.8706 | 0.6 |  | 365.2186 | 5.4 |  | 513.3026 | 1.8 |  | 480.281 | 2.4 |
| 270.1456 | 0.7 |  | 307.9943 | 1.6 |  | 472.8318 | 0.5 |  | 363.1910 | 1.3 |  | 496.2773 | 1.4 |  | 479.298 | 1.2 |
| 243.1462 | 0.8 |  | 294.3419 | 1.4 |  | 442.6534 | 0.5 |  | 297.1554 | 1.5 |  | 495.2936 | 1.6 |  | 474.159 | 0.9 |
| 243.1326 | 1.2 |  | 287.1714 | 10.0 |  | 435.7206 | 0.5 |  | 271.1766 | 20.0 |  | 477.2806 | 0.6 |  | 472.789 | 1.1 |
| 228.1350 | 0.8 |  | 276.5113 | 1.4 |  | 399.2238 | 0.9 |  | 269.1634 | 1.6 |  | 474.1738 | 0.7 |  | 383.228 | 1.2 |
| 226.1182 | 3.8 |  | 269.1612 | 8.7 |  | 381.2012 | 9.6 |  | 269.1462 | 3.5 |  | 399.2230 | 1.7 |  | 381.202 | 9.6 |
| 225.1224 | 0.7 |  | 267.9988 | 1.9 |  | 363.1919 | 1.6 |  | 254.1499 | 46.4 |  | 381.2015 | 13.1 |  | 367.147 | 0.9 |
| 210.1231 | 0.8 |  | 254.1506 | 2.8 |  | 360.6646 | 0.5 |  | 253.1658 | 11.0 |  | 363.1935 | 0.6 |  | 365.218 | 9.8 |
| 208.1083 | 1.4 |  | 249.9879 | 3.9 |  | 357.2144 | 0.6 |  | 252.1348 | 0.9 |  | 341.2188 | 1.6 |  | 363.192 | 1.1 |
| 208.0963 | 1.3 |  | 214.1791 | 1.3 |  | 349.0199 | 0.6 |  | 251.1385 | 1.1 |  | 339.2042 | 1.5 |  | 339.192 | 1.4 |
| 207.1246 | 0.6 |  | 211.1439 | 1.8 |  | 339.2004 | 1.5 |  | 236.1387 | 1.4 |  | 324.1917 | 3.3 |  | 324.193 | 0.7 |
| 199.0990 | 0.5 |  | 210.1239 | 3.9 |  | 322.1640 | 0.6 |  | 227.1398 | 0.8 |  | 323.2079 | 2.5 |  | 323.209 | 1.1 |
| 198.1123 | 1.1 |  | 199.1078 | 1.7 |  | 321.1793 | 0.5 |  | 223.1446 | 0.9 |  | 322.1663 | 1.0 |  | 322.167 | 0.7 |
| 195.1124 | 0.6 |  | 193.0970 | 3.6 |  | 314.8150 | 0.5 |  | 212.1390 | 2.2 |  | 321.1913 | 1.1 |  | 321.180 | 1.2 |
| 193.0970 | 1.2 |  | 184.6905 | 1.9 |  | 287.1726 | 5.4 |  | 210.1244 | 0.8 |  | 312.7150 | 0.6 |  | 297.156 | 2.2 |
| 192.1141 | 0.6 |  | 180.1013 | 1.3 |  | 285.1572 | 0.9 |  | 209.1282 | 0.8 |  | 305.1977 | 0.7 |  | 287.161 | 1.2 |
| 191.1024 | 0.6 |  | 173.0919 | 27.5 |  | 270.1451 | 12.8 |  | 199.1073 | 1.4 |  | 292.4295 | 0.6 |  | 285.147 | 0.7 |
| 184.7629 | 0.9 |  | 169.8066 | 1.2 |  | 269.1621 | 2.5 |  | 195.1129 | 11.2 |  | 287.1743 | 6.7 |  | 271.176 | 19.1 |
| 180.1017 | 0.7 |  | 168.1017 | 14.4 |  | 269.1490 | 3.3 |  | 194.1288 | 1.2 |  | 270.1452 | 25.2 |  | 270.651 | 0.6 |
| 179.8684 | 0.5 |  | 164.1068 | 4.9 |  | 251.1389 | 1.7 |  | 193.0973 | 1.2 |  | 269.1623 | 3.6 |  | 270.133 | 1.5 |
| 173.0918 | 36.2 |  | 157.0969 | 14.4 |  | 242.0207 | 0.5 |  | 182.1172 | 8.4 |  | 269.1491 | 5.1 |  | 269.150 | 6.9 |
| 155.0813 | 4.0 |  | 155.0816 | 3.7 |  | 228.1340 | 3.8 |  | 175.1075 | 2.6 |  | 268.1650 | 1.1 |  | 255.144 | 0.9 |
| 154.4425 | 0.5 |  | 152.1069 | 1.8 |  | 227.1511 | 0.7 |  | 173.0921 | 13.2 |  | 253.1297 | 1.0 |  | 254.150 | 54.5 |
| 145.0969 | 4.1 |  | 139.0865 | 2.1 |  | 227.1387 | 1.7 |  | 164.1070 | 2.6 |  | 252.1339 | 0.8 |  | 253.166 | 11.7 |
| 131.0813 | 100.0 |  | 131.0814 | 100.0 |  | 226.1182 | 1.7 |  | 158.0807 | 1.0 |  | 251.1390 | 2.4 |  | 251.138 | 3.9 |
| 128.0703 | 4.3 |  | 129.1021 | 6.0 |  | 223.1439 | 0.5 |  | 157.0971 | 33.9 |  | 228.1342 | 5.6 |  | 241.215 | 0.9 |
| 127.0865 | 3.8 |  | 128.0704 | 1.9 |  | 211.1083 | 0.5 |  | 155.0817 | 1.5 |  | 227.1388 | 2.5 |  | 238.119 | 1.5 |
| 119.5985 | 0.5 |  | 127.0867 | 2.7 |  | 210.1235 | 5.6 |  | 146.8862 | 0.7 |  | 223.1445 | 1.2 |  | 236.139 | 1.0 |
| 114.0548 | 22.5 |  | 126.0443 | 1.2 |  | 209.1395 | 0.8 |  | 145.8965 | 0.9 |  | 216.2471 | 0.5 |  | 234.374 | 0.8 |
| 114.0497 | 1.1 |  | 120.9156 | 1.3 |  | 209.1284 | 0.6 |  | 139.0867 | 5.7 |  | 215.1020 | 0.6 |  | 227.139 | 2.6 |
| 113.0705 | 10.0 |  | 116.0707 | 1.5 |  | 195.1129 | 5.1 |  | 131.0815 | 42.6 |  | 212.1394 | 4.2 |  | 223.144 | 1.5 |
| 113.0596 | 67.3 |  | 115.0866 | 9.9 |  | 193.0972 | 1.7 |  | 129.1020 | 3.3 |  | 210.1236 | 6.9 |  | 222.248 | 0.8 |
| 112.0756 | 1.5 |  | 114.0627 | 1.7 |  | 192.1129 | 1.4 |  | 127.0868 | 0.8 |  | 210.1120 | 0.8 |  | 212.139 | 3.1 |
| 110.0598 | 1.1 |  | 114.0549 | 24.2 |  | 182.1275 | 0.7 |  | 115.0865 | 33.9 |  | 209.1284 | 1.4 |  | 209.128 | 1.6 |
| 103.6100 | 0.5 |  | 113.0704 | 8.5 |  | 182.1174 | 11.9 |  | 114.0550 | 7.7 |  | 195.1127 | 17.1 |  | 204.999 | 0.9 |
| 97.7251 | 0.9 |  | 113.0596 | 84.5 |  | 175.1078 | 3.4 |  | 113.0706 | 3.0 |  | 194.6992 | 0.6 |  | 201.087 | 0.8 |
| 96.0482 | 0.7 |  | 112.0756 | 43.9 |  | 173.0919 | 28.6 |  | 113.0597 | 100.0 |  | 194.1291 | 1.6 |  | 199.107 | 1.1 |
| 96.0443 | 13.6 |  | 112.0717 | 1.0 |  | 168.1022 | 0.6 |  | 112.0757 | 20.2 |  | 193.0970 | 1.7 |  | 195.113 | 12.6 |
| 95.0490 | 17.7 |  | 98.0251 | 1.3 |  | 167.0807 | 0.5 |  | 111.0917 | 0.9 |  | 192.1132 | 2.1 |  | 194.129 | 2.2 |
| 86.0599 | 21.5 |  | 96.0443 | 14.1 |  | 165.0658 | 0.6 |  | 110.0601 | 1.9 |  | 187.9796 | 0.6 |  | 192.547 | 0.6 |
| 85.0759 | 1.4 |  | 95.0491 | 22.3 |  | 164.1067 | 3.3 |  | 107.3057 | 0.8 |  | 182.1174 | 17.1 |  | 183.076 | 1.1 |
| 85.0646 | 8.5 |  | 91.8350 | 1.3 |  | 158.0812 | 1.5 |  | 100.2857 | 0.7 |  | 175.1078 | 4.5 |  | 182.117 | 19.6 |
| 82.1172 | 0.6 |  | 89.2612 | 1.2 |  | 157.0970 | 41.2 |  | 96.0443 | 12.5 |  | 173.0920 | 24.8 |  | 175.108 | 4.4 |
| 79.0451 | 0.5 |  | 86.0963 | 2.3 |  | 155.0815 | 3.6 |  | 95.0491 | 30.6 |  | 171.5368 | 0.6 |  | 166.123 | 0.8 |
|  |  |  | 86.0600 | 15.5 |  | 152.1069 | 0.6 |  | 86.0600 | 7.3 |  | 167.0811 | 1.4 |  | 165.554 | 0.7 |
|  |  |  | 85.0648 | 12.5 |  | 147.2195 | 0.4 |  | 85.0647 | 15.5 |  | 164.1071 | 5.1 |  | 164.761 | 0.7 |
|  |  |  | 84.0809 | 2.1 |  | 145.0974 | 2.6 |  | 85.0618 | 0.4 |  | 158.0815 | 2.9 |  | 164.107 | 5.3 |
|  |  |  | 77.0431 | 1.3 |  | 140.0705 | 1.1 |  | 83.0283 | 0.7 |  | 157.0971 | 59.1 |  | 159.556 | 0.7 |
|  |  |  |  |  |  | 139.0864 | 6.3 |  | 82.0647 | 0.7 |  | 156.6254 | 0.6 |  | 158.081 | 2.2 |
|  |  |  |  |  |  | 138.0547 | 0.5 |  | 78.8612 | 0.7 |  | 155.0814 | 2.0 |  | 157.097 | 59.9 |
|  |  |  |  |  |  | 135.0919 | 0.5 |  | 77.0579 | 0.6 |  | 152.1071 | 1.9 |  | 152.107 | 1.7 |
|  |  |  |  |  |  | 131.0814 | 38.4 |  | 71.0491 | 2.2 |  | 145.0970 | 1.7 |  | 147.549 | 0.6 |

**Table S7:** MS/MS fragmentation spectra used to assign Coprogen and corresponding degradation products in Figure 5.

| **#31** | |  | **#32** | |  | **#33** | |  | **#34** | |
| --- | --- | --- | --- | --- | --- | --- | --- | --- | --- | --- |
| Precursor *m/z* | 769.3963 |  | Precursor *m/z* | 753.4017 |  | Precursor *m/z* | 737.4067 |  | Precursor *m/z* | 721.4119 |
| Fragment *m/z* | Intensity (%) |  | Fragment *m/z* | Intensity (%) |  | Fragment *m/z* | Intensity (%) |  | Fragment *m/z* | Intensity (%) |
| 756.4151 | 0.7 |  | 740.9423 | 1.0 |  | 738.866 | 1.2 |  | 738.7526 | 1.6 |
| 740.8502 | 0.9 |  | 738.9820 | 1.2 |  | 625.354 | 6.5 |  | 721.3346 | 2.8 |
| 738.7735 | 1.1 |  | 696.5430 | 1.2 |  | 607.347 | 6.1 |  | 703.4001 | 1.0 |
| 738.7033 | 1.0 |  | 642.1543 | 0.9 |  | 513.303 | 5.3 |  | 609.3603 | 20.9 |
| 618.4381 | 0.7 |  | 641.3498 | 1.8 |  | 496.278 | 2.5 |  | 592.3346 | 1.6 |
| 472.8155 | 0.7 |  | 623.3401 | 1.2 |  | 495.293 | 6.8 |  | 591.3498 | 17.1 |
| 472.7774 | 0.8 |  | 529.2983 | 2.9 |  | 478.265 | 1.0 |  | 573.3394 | 0.9 |
| 470.9662 | 0.6 |  | 511.2876 | 4.0 |  | 474.214 | 0.9 |  | 550.3229 | 2.1 |
| 397.1984 | 1.4 |  | 397.1978 | 1.4 |  | 472.875 | 0.8 |  | 549.3411 | 0.9 |
| 355.1981 | 4.0 |  | 392.5018 | 1.2 |  | 472.836 | 1.0 |  | 498.8751 | 0.7 |
| 285.1451 | 1.8 |  | 381.2010 | 4.4 |  | 460.256 | 0.7 |  | 497.3072 | 2.8 |
| 270.1355 | 0.9 |  | 363.1905 | 1.4 |  | 453.271 | 1.3 |  | 480.2807 | 7.7 |
| 267.1337 | 0.7 |  | 357.2130 | 5.6 |  | 436.254 | 1.4 |  | 479.2968 | 6.8 |
| 243.1466 | 3.6 |  | 354.2027 | 1.0 |  | 435.260 | 1.7 |  | 472.8552 | 0.7 |
| 243.1335 | 1.6 |  | 344.2158 | 1.0 |  | 419.229 | 1.1 |  | 462.2706 | 1.4 |
| 241.2244 | 0.9 |  | 340.1877 | 1.0 |  | 418.244 | 1.7 |  | 453.2699 | 1.9 |
| 241.1680 | 0.7 |  | 339.2026 | 8.4 |  | 381.202 | 9.0 |  | 437.1891 | 2.4 |
| 239.1391 | 1.2 |  | 322.1767 | 1.8 |  | 381.128 | 0.7 |  | 435.2605 | 4.0 |
| 228.1344 | 1.1 |  | 321.1908 | 1.1 |  | 377.166 | 0.7 |  | 403.2437 | 1.0 |
| 226.1186 | 3.7 |  | 287.1609 | 1.6 |  | 363.192 | 1.6 |  | 400.0091 | 0.8 |
| 225.1231 | 1.1 |  | 285.1454 | 1.5 |  | 357.213 | 1.3 |  | 381.2018 | 12.3 |
| 214.5164 | 0.6 |  | 279.8900 | 0.9 |  | 354.201 | 0.7 |  | 367.1470 | 1.5 |
| 208.1315 | 0.7 |  | 269.1493 | 3.1 |  | 346.248 | 0.8 |  | 363.1930 | 1.0 |
| 208.1083 | 2.4 |  | 257.6616 | 1.1 |  | 344.071 | 0.8 |  | 341.2183 | 13.5 |
| 208.0969 | 1.1 |  | 245.1606 | 1.5 |  | 341.218 | 7.4 |  | 339.1920 | 1.8 |
| 207.1247 | 1.2 |  | 239.1389 | 0.9 |  | 340.187 | 0.8 |  | 324.1917 | 24.5 |
| 198.1128 | 1.3 |  | 236.0656 | 0.9 |  | 339.202 | 4.4 |  | 323.2079 | 15.3 |
| 197.1283 | 1.1 |  | 228.1338 | 9.3 |  | 324.191 | 11.9 |  | 319.2365 | 0.8 |
| 192.1135 | 1.0 |  | 227.1505 | 4.3 |  | 323.208 | 8.0 |  | 306.1803 | 3.7 |
| 184.6870 | 0.9 |  | 227.1385 | 2.2 |  | 322.177 | 1.3 |  | 305.1980 | 1.8 |
| 180.1019 | 2.0 |  | 210.1232 | 9.0 |  | 314.639 | 0.7 |  | 287.1600 | 2.7 |
| 173.0920 | 28.4 |  | 209.1277 | 2.2 |  | 306.181 | 1.8 |  | 284.1886 | 0.9 |
| 157.0983 | 0.6 |  | 197.1288 | 1.4 |  | 305.197 | 1.0 |  | 270.1338 | 2.2 |
| 155.0815 | 4.1 |  | 195.1122 | 5.4 |  | 287.160 | 1.4 |  | 269.1496 | 5.7 |
| 145.9255 | 0.7 |  | 193.0969 | 3.5 |  | 285.144 | 0.9 |  | 260.3320 | 0.9 |
| 140.0711 | 0.6 |  | 192.1134 | 4.5 |  | 269.150 | 4.3 |  | 255.0370 | 0.8 |
| 131.0815 | 100.0 |  | 182.1174 | 7.6 |  | 251.139 | 1.7 |  | 251.1393 | 4.3 |
| 128.0704 | 0.9 |  | 180.1015 | 1.5 |  | 245.161 | 0.8 |  | 241.2253 | 1.0 |
| 127.0867 | 1.4 |  | 175.1079 | 2.2 |  | 241.249 | 0.9 |  | 238.1185 | 2.2 |
| 122.2854 | 0.7 |  | 173.0919 | 15.8 |  | 238.118 | 1.1 |  | 229.1663 | 1.4 |
| 118.1990 | 0.6 |  | 167.0815 | 1.3 |  | 229.165 | 1.0 |  | 227.1391 | 3.2 |
| 116.8224 | 0.6 |  | 164.1069 | 2.7 |  | 228.134 | 5.9 |  | 223.1439 | 1.5 |
| 114.0629 | 1.1 |  | 158.0809 | 1.3 |  | 227.139 | 2.8 |  | 212.1393 | 21.5 |
| 114.0549 | 26.0 |  | 157.0970 | 19.7 |  | 212.139 | 9.7 |  | 211.1549 | 3.1 |
| 113.0705 | 10.2 |  | 155.0814 | 2.9 |  | 211.155 | 2.7 |  | 209.1283 | 2.3 |
| 113.0597 | 91.8 |  | 139.0864 | 2.5 |  | 210.124 | 4.6 |  | 202.1264 | 2.7 |
| 112.0757 | 2.7 |  | 132.8399 | 1.0 |  | 209.140 | 0.9 |  | 201.1232 | 4.6 |
| 110.0600 | 1.4 |  | 132.0844 | 1.0 |  | 209.129 | 3.3 |  | 199.2635 | 0.7 |
| 100.7364 | 0.6 |  | 131.0814 | 60.5 |  | 198.197 | 0.7 |  | 195.1127 | 35.9 |
| 96.0444 | 16.0 |  | 127.0865 | 2.1 |  | 195.113 | 20.5 |  | 194.1289 | 6.0 |
| 95.0491 | 21.2 |  | 115.0865 | 30.3 |  | 194.129 | 5.2 |  | 184.6911 | 0.9 |
| 86.0600 | 20.4 |  | 114.0549 | 13.0 |  | 193.097 | 2.7 |  | 184.6802 | 0.9 |
| 85.0760 | 2.5 |  | 113.0707 | 3.8 |  | 192.113 | 1.3 |  | 182.1173 | 15.6 |
| 85.0647 | 13.3 |  | 113.0596 | 100.0 |  | 184.708 | 1.0 |  | 181.1333 | 1.3 |
|  |  |  | 112.0757 | 2.6 |  | 182.117 | 11.4 |  | 175.1077 | 5.3 |

**Table S8:** MS/MS fragmentation spectra used to assign Dimerum acid and corresponding degradation products in Figure 5.

| **#41** | |  | **#42** | |  | **#43** | |
| --- | --- | --- | --- | --- | --- | --- | --- |
| Precursor *m/z* | 485.2598 |  | Precursor *m/z* | 469.2648 |  | Precursor *m/z* | 453.2698 |
| Fragment *m/z* | Intensity (%) |  | Fragment *m/z* | Intensity (%) |  | Fragment *m/z* | Intensity (%) |
| 399.9299 | 0.3 |  | 357.2134 | 3.4 |  | 386.2499 | 3.1 |
| 373.2086 | 0.5 |  | 340.1882 | 0.3 |  | 385.2449 | 2.2 |
| 355.1975 | 3.0 |  | 339.2027 | 8.9 |  | 341.2181 | 50.8 |
| 252.6773 | 0.3 |  | 324.1918 | 0.8 |  | 335.1599 | 20.4 |
| 243.1463 | 4.5 |  | 322.1999 | 0.4 |  | 331.0270 | 1.9 |
| 243.1302 | 1.4 |  | 321.1922 | 0.6 |  | 324.1916 | 40.9 |
| 228.1342 | 1.3 |  | 309.1917 | 0.4 |  | 323.2077 | 73.2 |
| 226.1417 | 0.4 |  | 273.5017 | 0.3 |  | 321.2375 | 1.9 |
| 226.1186 | 3.1 |  | 259.4561 | 0.3 |  | 320.2376 | 10.0 |
| 225.1354 | 0.6 |  | 245.1611 | 1.5 |  | 319.2331 | 14.2 |
| 225.1231 | 0.8 |  | 243.1339 | 0.6 |  | 306.1813 | 5.2 |
| 210.1240 | 1.6 |  | 241.2530 | 0.4 |  | 305.1962 | 3.2 |
| 209.1388 | 0.5 |  | 236.4974 | 0.3 |  | 298.4440 | 1.9 |
| 208.1080 | 2.2 |  | 228.1342 | 2.5 |  | 285.1445 | 3.3 |
| 207.6415 | 0.3 |  | 227.1504 | 5.8 |  | 283.1285 | 3.7 |
| 207.1241 | 0.4 |  | 227.1385 | 1.5 |  | 282.1458 | 1.8 |
| 195.1127 | 0.5 |  | 212.1393 | 0.7 |  | 268.1376 | 12.7 |
| 193.0969 | 0.7 |  | 210.1236 | 5.0 |  | 267.1336 | 21.0 |
| 192.1131 | 1.3 |  | 209.1399 | 0.9 |  | 255.1458 | 2.3 |
| 191.1289 | 0.4 |  | 209.1283 | 1.2 |  | 238.1184 | 11.3 |
| 184.7002 | 0.5 |  | 199.1441 | 0.5 |  | 237.1339 | 4.7 |
| 181.0972 | 0.4 |  | 197.8588 | 0.3 |  | 236.1281 | 44.2 |
| 157.0604 | 0.3 |  | 195.1128 | 2.5 |  | 234.6301 | 4.7 |
| 155.0814 | 0.3 |  | 194.1282 | 0.7 |  | 229.1658 | 8.9 |
| 150.0785 | 0.4 |  | 193.1208 | 0.3 |  | 227.1386 | 10.8 |
| 137.6273 | 0.3 |  | 193.0972 | 5.1 |  | 226.1910 | 2.1 |
| 131.0815 | 7.2 |  | 192.1130 | 4.8 |  | 212.1393 | 34.9 |
| 118.0647 | 0.4 |  | 191.1289 | 0.5 |  | 211.1550 | 28.0 |
| 114.0549 | 7.7 |  | 184.7101 | 0.3 |  | 210.1681 | 4.4 |
| 113.0597 | 100.0 |  | 182.1175 | 3.3 |  | 210.1490 | 3.6 |
| 97.6942 | 0.4 |  | 165.1019 | 0.8 |  | 209.1649 | 5.8 |
| 97.0760 | 0.4 |  | 164.1179 | 0.5 |  | 209.1283 | 6.5 |
| 96.0442 | 0.5 |  | 132.4349 | 0.2 |  | 202.1269 | 13.9 |
| 95.0491 | 10.7 |  | 131.7037 | 0.3 |  | 201.1234 | 47.6 |
| 86.8299 | 0.3 |  | 131.0815 | 1.1 |  | 195.1127 | 57.8 |
| 86.0632 | 0.6 |  | 115.0866 | 10.5 |  | 194.1287 | 25.9 |
| 86.0600 | 6.3 |  | 114.0550 | 0.3 |  | 193.1443 | 1.9 |
| 85.0760 | 2.4 |  | 113.0597 | 100.0 |  | 186.1125 | 2.2 |
| 85.0648 | 20.8 |  | 107.4073 | 0.3 |  | 185.1280 | 7.8 |
| 83.0493 | 0.3 |  | 106.4298 | 0.3 |  | 185.1142 | 3.7 |
| 74.3880 | 0.4 |  | 97.6979 | 0.4 |  | 184.7188 | 2.3 |
| 71.0491 | 5.3 |  | 96.2303 | 0.3 |  | 184.6124 | 5.5 |
| 70.4132 | 0.3 |  | 95.0491 | 11.0 |  | 184.0965 | 4.7 |
| 70.0651 | 6.3 |  | 86.0600 | 1.0 |  | 183.1130 | 9.9 |
| 69.9454 | 0.3 |  | 85.0759 | 0.3 |  | 182.1173 | 15.9 |
| 69.0336 | 0.5 |  | 85.0647 | 19.1 |  | 169.1053 | 20.4 |
| 68.0494 | 3.8 |  | 72.0345 | 0.3 |  | 168.1018 | 100.0 |
| 67.0542 | 18.4 |  | 71.0491 | 5.3 |  | 166.1055 | 2.8 |
| 65.0385 | 0.3 |  | 70.0675 | 0.5 |  | 165.1021 | 8.1 |
| 60.3052 | 0.4 |  | 70.0651 | 9.9 |  | 161.0135 | 2.0 |
| 58.9339 | 0.3 |  | 69.0334 | 0.5 |  | 155.0897 | 2.1 |
| 58.3311 | 0.2 |  | 68.0495 | 0.5 |  | 154.0861 | 7.3 |
| 57.0698 | 2.8 |  | 67.1709 | 0.3 |  | 140.2842 | 1.8 |
| 54.4242 | 0.2 |  | 67.0542 | 16.5 |  |  |  |


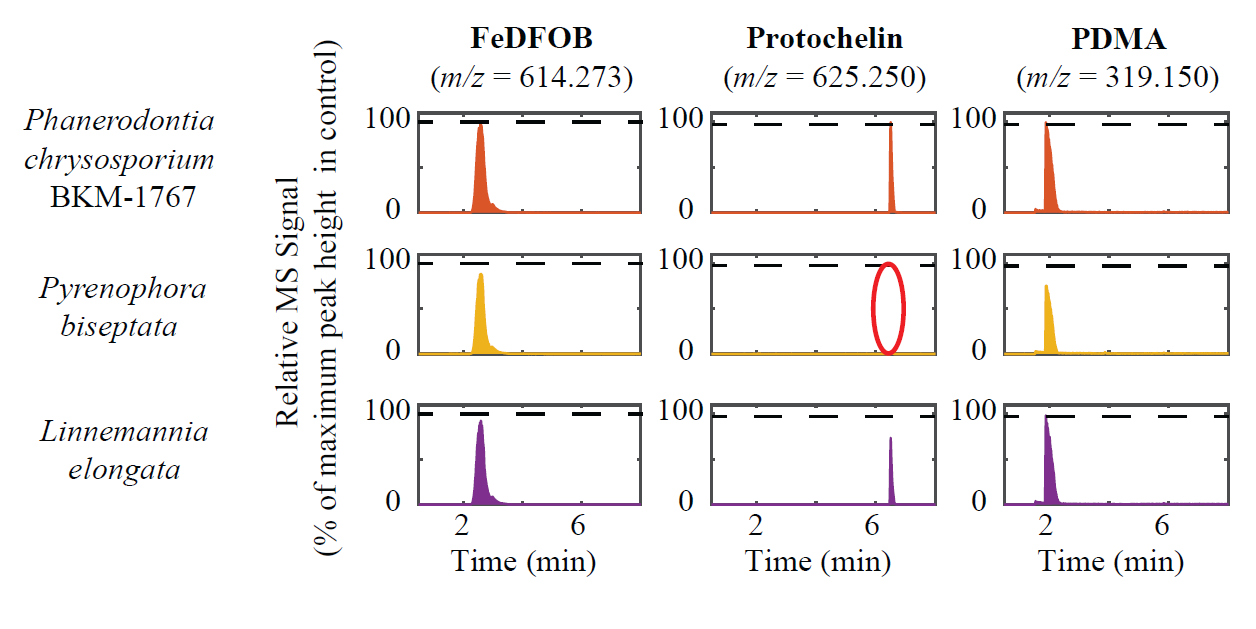


**Figure S1:** LC-MS peaks showing dissolved siderophores after incubation in iron-replete MSB medium for mycelium of each of the four fungi after 2 d in comparison to initial peak heights (dashed line). To create Fe replete conditions, 300µM of FeCl3 was added during fungal incubation. This concentration exceeded the combined concentration of Fe chelators ([EDTA] = 100µM and each of the three siderophores added at 6.7 µM). Shown is the FeDFOB peak because in iron replete conditions, DFOB was fully bound to iron and free unbound DFOB was negligible. Protochelin and PDMA were measured as the free siderophore under the LC-MS conditions which included an acidic mobile phase buffer (0.1% formic acid).


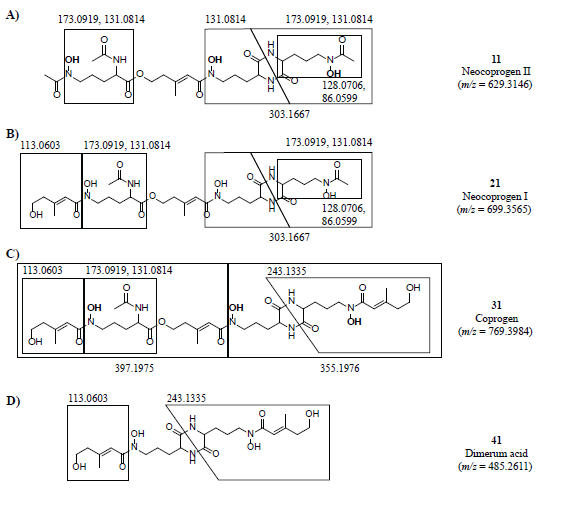


**Figure S2:** Structures and characteristic MS/MS fragments of the four siderophores with the highest peak area produced by *P. biseptata*: A) Neocoprogen II, B) Neocoprogen I, C) Coprogen, D) Dimerum acid. Shown are the apo forms of each of the siderophores.

**

**Figure S3:** MS/MS fragment *m/z* values in red were used to assign the position of hydroxamate reduction (Figure 5, Table S3) in the four major siderophores produced by *P. biseptata*: A) Neocoprogen II, B) Neocoprogen I, C) Coprogen, D) Dimerum acid.

**

**Figure S4:** LC-MS chromatograms for the DFOB-O (m/z = 545.4) degradation product after 72h incubation of *S. cerevisiae* mycelium with DFOB in iron-limited media (-Fe) and iron-replete media (+Fe) and comparison to a sterile control.
